# Development and characterization of a camelid derived antibody targeting a linear epitope in the hinge domain of human PCSK9 protein

**DOI:** 10.1038/s41598-022-16453-3

**Published:** 2022-07-16

**Authors:** Xinyang Li, Jun Hong, Xiaoyan Gao, Meiniang Wang, Naibo Yang

**Affiliations:** 1 College of Life Science and Engineering, Henan University of Urban Construction, Pingdingshan, 467036 China; 2 BGI-Shenzhen, Shenzhen, 518083 China; 3 Complete Genomics, Inc., 2904 Orchard Parkway, San Jose, CA 95134 USA

**Keywords:** Drug discovery, Biologics, Antibody fragment therapy

## Abstract

PCSK9 is an effective target for lowering LDL-c. Previously, a camelid-human chimeric heavy chain antibody VHH-B11-Fc targeting human PCSK9 was designed. It had a potent hypolipidemic effect. However, the nanobody VHH-B11 interacts with PCSK9 at low affinity, while camelid VHH exhibits some immunogenicity. Moreover, the interacting epitope is yet to be identified, although VHH-B11 was shown to have distinct hPCSK9-binding epitopes for Evolocumab. This might impede the molecule’s progress from bench to bedside. In the present study, we designed various configurations to improve the affinity of VHH-B11 with hPCSK9 (< 10 nM) that in turn enhanced the druggability of VHH-B11-Fc. Then, 17 amino acids were specifically mutated to increase the degree of humanization of the nanobody VHH-B11. Using phage display and sequencing technology, the linear epitope “STHGAGW” (amino acids 447–452) was identified in the hinge region of PCSK9 as the interacting site between VHH-B11-Fc and hPCSK9. Unlike the interaction epitope of Evolocumab, located in the catalytic region of PCSK9, the binding epitope of VHH-B11 is located in the hinge region of PCSK9, which is rarely reported. These findings indicated that a specific mechanism underlying this interaction needs to be explored.

## Introduction

Cardiovascular and cerebrovascular diseases refer to the general term for some diseases, including coronary heart disease, myocardial infarction, cerebral apoplexy, myocarditis, and other ischemic or hemorrhagic diseases caused by the following symptoms: hyperlipidemia, atherosclerosis, and/or hypertension^1^. Despite adopting the most advanced treatments for cardiovascular and cerebrovascular diseases, > 50% of survivors of these diseases are unable to care for themselves and the quality of life declines severely. Previous studies have reported that cardiovascular disease has a mortality of up to 15 million people each year, surpassing cancer as the leading cause of death^2–4^. Presently, 3-hydroxy-3-methylglutaryl-coA reductase inhibitor is the first-line clinical lipid-lowering statin. However, recent studies have shown that some patients will develop drug tolerance if statin is administered for a prolonged time; then, the treatment needs to be discontinued, seeking other lipid-lowering approaches^1,2,5^.

An effective and safe drug is the monoclonal antibody (Ab)^6,7^. In a previous study, we designed and developed a llama-human chimera heavy chain VHH-B11-Fc Ab, which showed similar lipid-lowering effects to the approved Evolocumab drug in hepatoma cells and human *proprotein convertase subtilisin/kexin* 9 (*hPCSK9*) transgenic rat model^7^. However, the druggability of this Ab is not excellent, which is manifested as inadequate affinity (> 0.1 nM), immunogenicity (camelid variable region) and unknown epitopes. Therefore, the present study aimed to improve the druggability of this potential drug molecule in the following aspects. The affinity of therapeutic Ab should be ≤ 0.1 nM. Hence, we attempted to improve the affinity between the VHH-B11 Ab and PCSK9. For instance, the transformation of fragmentary Ab from monovalent to polyvalent effectively improves the affinity and function^8–10^. This is mainly achieved by short peptide ligation (“Cablivi” from Ablynx Inc.) and Fc fusion (“KN035” from Alphamab Oncology Inc.)^11–13^. Secondly, good therapeutic Abs should have low immunogenicity. The high immunogenicity may cause major side effects^14^. For instance, Pfizer had discontinued the global development of bococizumab because of its high immunogenicity in 2016^15^. In this study, the humanization degree of the VHH-B11 was improved by replacing some hot spot amino acids^16^. Finally, we identified the interaction epitope between the VHH-B11-Fc Ab and human PCSK9 (hPCSK9) based on sequencing, phage display, and random polypeptide library technologies. All in a word, the present study aimed to develop and characterize a camelid derived Ab (VHH-B11-Fc) reported previously.

## Results

### Monovalent nanobody VHH-B11 expression in different systems

**Figure 1:**
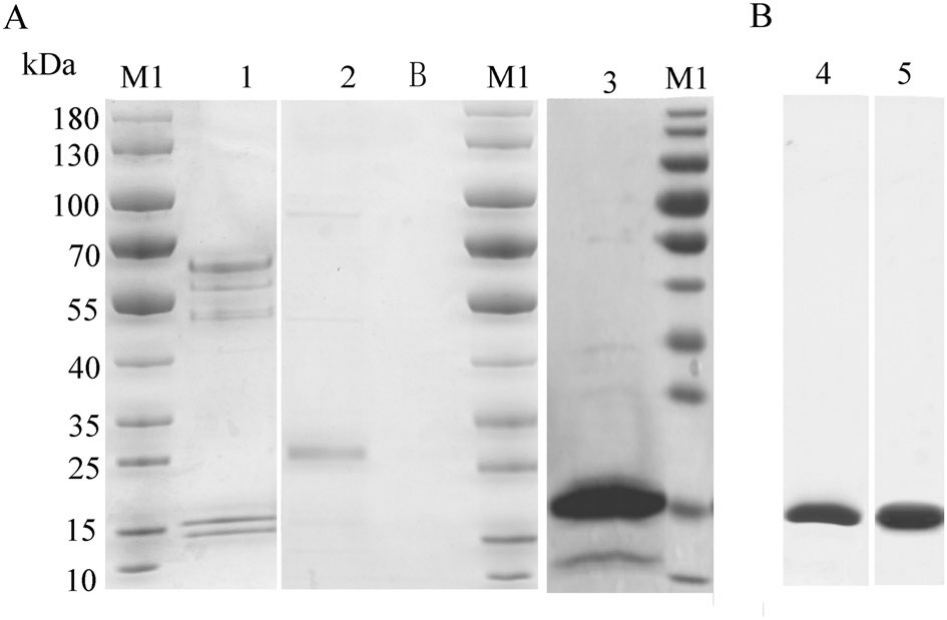
The SDS–PAGE gel of the VHH-B11. M1: the protein marker (Fermentas, USA). (**A**) Lane 1: the VHH-B11 antibody expressed by BL21 *E. coli* (*pET28a*); lane 2: the VHH-B11 expressed by TG1 *E. coli* (*pMECS*); lane B: blank control; lane 3: the VHH-B11 expressed by HB2151 *E. coli* (*pMECS*); (**B**) lane 4: the VHH-B11 expressed by *P. pastoris* X33 (*pPICZα*); lane 5: the VHH-B11 expressed by HEK293F (*pCDNA3.4*).

**Figure 2:**
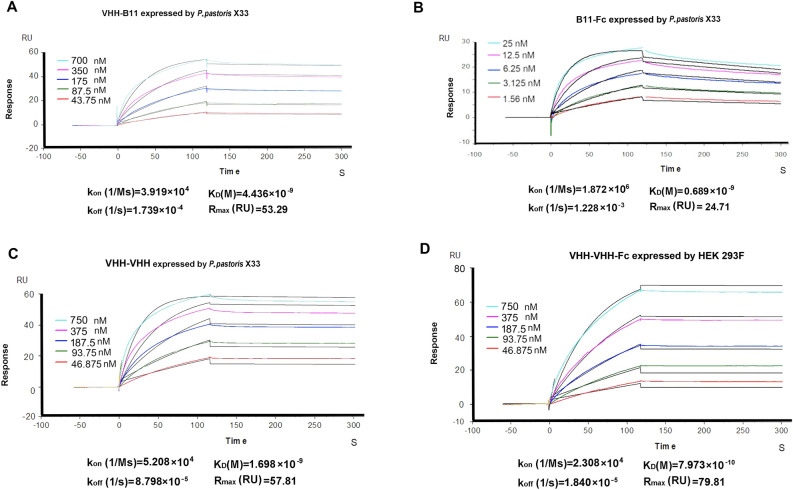
Affinity tests between the VHH-B11 or its derived antibodies and human PCSK9. The affinity was determined by SPR technology. Taking VHH-B11 for example, two-fold dilution of the VHH-B11 Ab (five concentrations in nM) expressed by *P. pastoris* X33 were loaded to bind the hPCSK9 coated on the CM5 chip. Each colored line represents a on concentration (in nM) of the VHH-B11 Ab. The black line was the automatic fitting curve by the built-in evaluation software of Biacore T200. The horizontal axis represents the injection time, and the vertical axis represents the response unit (RU) value of the VHH-B11 interaction with hPCSK9. The VHH-B11 injection time point was set as 0 s by the evaluation software. (**A**) VHH-B11; (**B**) VHH-B11-Fc; (**C**) VHH-VHH; (**D**) VHH-VHH-Fc.

Multiple systems were adopted to express the VHH-B11 Ab to seek a cost-effective Ab preparation method, preferably with one-step affinity purification for later preparation. Briefly, VHH was expressed in three expression systems (*Escherichia coli* *(E. coli)*, *Pichia pastoris (P. pastoris)* and HEK293F mammalian cells). As shown in Fig. 1 and Figs. S1–S5, the VHH-B11 Ab was expressed successfully in the three different systems. VHH-B11 was expressed in BL21 *E. coli* (lane 1: *pET28a-VHH-B11*), TG1 *E. coli* (lane 2: *pMECS-VHH-B11*), and HB2151 *E. coli* (lane 3: *pMECS-VHH-B11*) (Fig. 1A and Figs. S1–S3). Due to the intracellular and low soluble expression in BL21 *E. coli* (*pET28a*, lane 1), adequate results by only one step Ni^2+^ affinity purification is difficult. Also, ultrasonication resulted in partial degradation of the VHH-B11 Ab. In the case of TG1 *E. coli* (lane 2: *pMECS-VHH-B11*), the undesired protein band (~ 30 kDa) appeared on the gel. Lane B is the blank control. In lane 3, HB2151 *E. coli* (*pMECS-VHH-B11*) also seems to be not very suitable for VHH protein expression. Because one-step Ni^2+^-affinity purification provided an unsatisfactory eluate. As shown in Fig. 1B and Figs. S4, S5, the monovalent nanobody VHH-B11 was expressed in *P. pastoris* (lane 4) and HEK293F mammalian cells (lane 5). Because of the secretory expression, a favorable purification was achieved in a single Ni^2+^ affinity purification step without lysis. Due to the amount of heteroprotein bands that may require multi-stage purification, the three *E. coli* expression systems are not our preferred choice. Besides, considering the competitive advantages of low cost and convenient purification, yeast secretion seems to be a more optimal VHH expression system than that of mammalian cells. Furthermore, the affinity between PCSK9 and VHH-B11 expressed by *P. pastoris* was determinated by surface plasmon resonance (SPR) technology. As shown in Fig. 2A, VHH-B11 had an interaction affinity with PCSK9 at 4.436 nM.

### Polyvalent configuration design and comparison

**Figure 3:**
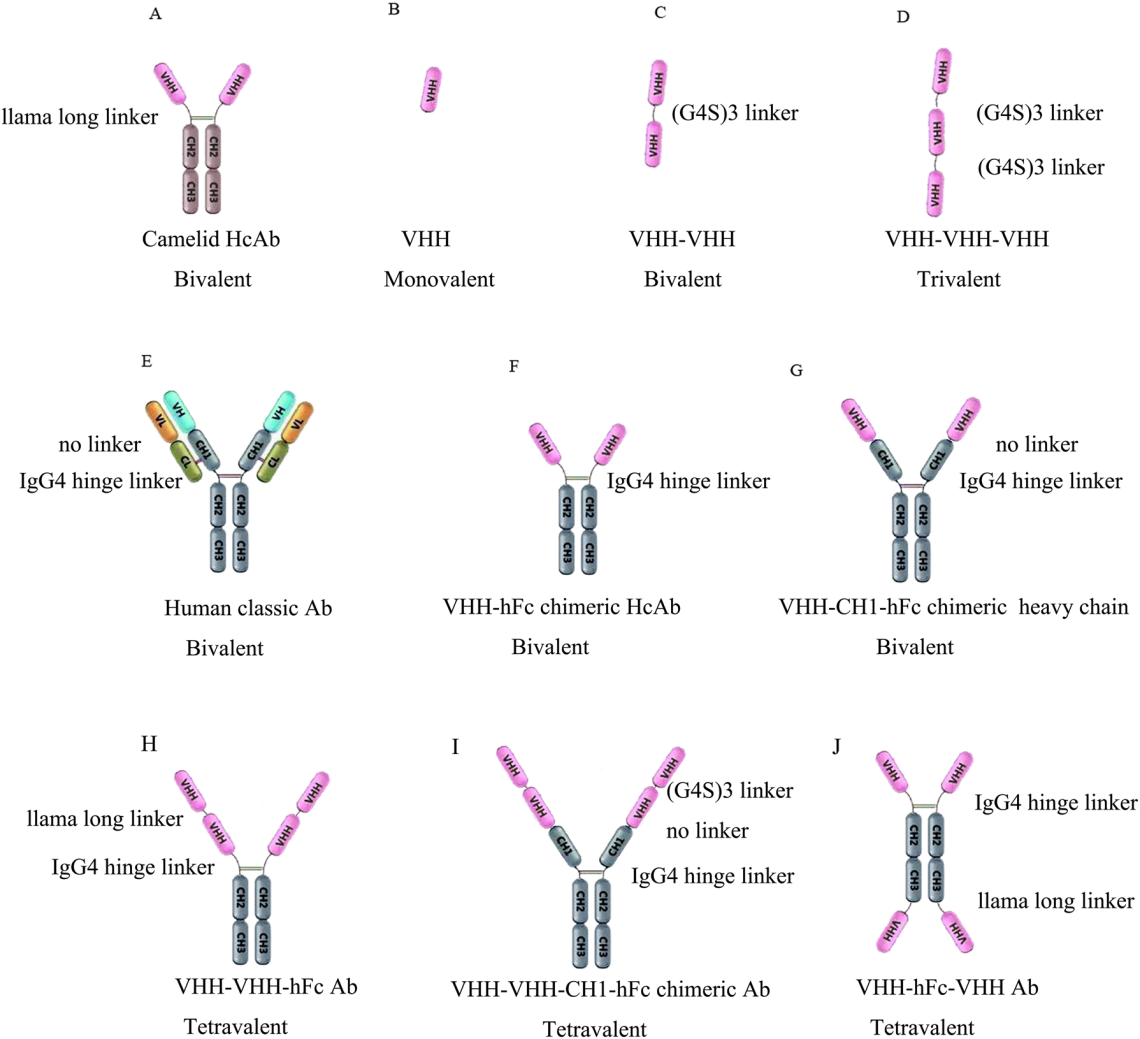
Schematic representation of Ab configurations. Schematic representation of different Abs’ configurations. (**A**) HcAb has its llama hinge linker: bivalent; (**B**) Camelid VHH: monovalent; (**C**) VHH-(G4S)3-VHH: bivalent; (**D**) VHH-(G4S)3-VHH-(G4S)3-VHH: trivalent; (**E**) Human classic Ab with its hinge linker: bivalent; (**F**) Llama VHH-human IgG4 hinge linker-IgG4 Fc chimeric HcAb: bivalent; (**G**) Llama VHH-human CH1-IgG4 hinge linker-IgG4 Fc chimeric heavy chain: bivalent; (**H**) VHH-llama long linker-VHH-human IgG4 hinge linker-IgG4 Fc: tetravalent; (**I**) VHH-(G4S)3 linker-VHH-human CH1-IgG4 hinge linker-IgG4 Fc chimeric Ab: tetravalent; (**J**) VHH-human IgG4 hinge linker-IgG4 Fc-llama long linker-VHH chimeric Ab: tetravalent. Thus, “(G4S)3” is a 15-peptide linker (amino acid sequence: GGGGSGGGGSGGGGS). “IgG4-hinge linker” means the hinge region between the human Fab and human IgG4 Fc (amino acid sequence: PPCPSCPAPEFLGGPS). “Llama long linker” means the hinge region between the llama Fab and llama IgG2 Fc (amino acid sequence: EPKIPQPQPKPQPQPQPQPKPQPKPEPECTCPKCP).

Figure 3A,E show the camelid heavy chain Ab (HcAb) and classic human Ab. The variable region of HcAb is also described as VHH (Fig. 3B). To increase the affinity and prolong the half-life of the VHH, we designed a series of VHH-based polyvalent Abs that can be divided into two classes of Ab configurations (Fig. 3). One was for non-IgG-like strategy (Fig. 3C,D) and the other for IgG-like strategy (Fig. 3F–J). However, both increased the affinity of VHH-B11 by > ten-fold or about 100-fold (Table 1). Peptide linker was used to form tandem bivalent or multivalent Abs for affinity increase. For instance, the GlyGlyGlyGlySerGlyGlyGlyGlySerGlyGlyGlyGlySer-(G4S)3 is the most common polypeptide linker with joint flexibility (Fig. 3C,D,I)^8,10^. “AlaAlaAla” is a representative of rigid peptides^11^ and the hinge region between Fab and Fc of the Ab is also considered a linker.

**Table 1:** Intercomparison of multiple VHH-B11-derived conformations.

Name	*E. coli*	*P. pastoris*	HEK293F	Affinity	Linker1; linker2; linker3	Relative expression level	Configuration diagram	Gel photo
VHH	✓	✓	✓	~ 4.4 nM	NA	High	Figure 3B	Figure 4, lane1
VHH-VHH		**✓**	**✓**	~ 1.7 nM	(G4S)3	High	Figure 3C	Figure 4, lane2
VHH-VHH-VHH			**✓**	~ 0.01 nM	(G4S)3; (G4S)3	High	Figure 3D	Figure 4, lane6
VHH-Fc		**✓**	**✓**	~ 0.6 nM	IgG4-hinge linker	High	Figure 3F	Figure 4, lane7
VHH-CH1-Fc			**✓**	NA	NA; IgG4-hinge linker	Very low	Figure 3G	NA
VHH-VHH-Fc			**✓**	~ 0.8 nM	Llama long linker; IgG4-hinge linker	Medium	Figure 3H	Figure 4, lane8
VHH-VHH-CH1-Fc			**✓**	NA	(G4S)3; NA; hIgG4-hinge linker	Low	Figure 3I	Figure 4, lane9
VHH-Fc-VHH			**✓**	~ 0.6 nM	IgG4-hinge linker; Llama long linker	Medium	Figure 3J	Figure 4, lane10

Note: “VHH” represents single domain Ab VHH-B11. “✓” indicates that the Ab is expressed in this expression system. “-” represents the linker between the domains (VHH, CH1 or Fc). “(G4S)3” is a 15-peptide linker (amino acid sequence: GGGGSGGGGSGGGGS). “IgG4-hinge linker” indicates the hinge region between human Fab and human IgG4 Fc (amino acid sequence: PPCPSCPAPEFLGGPS); “Llama long linker” represents the hinge region between llama Fab and llama IgG2 Fc (amino acid sequence: EPKIPQPQPKPQPQPQPQPKPQPKPEPECTCPKCP). Relative expression level represents the expression status of each Ab in HEK293F cell expression system under same conditions. “High” means the purified Ab expression level > 20 mg/mL; “medium” means ~ 10 mg/mL; “low” means < 1 mg/mL; “very low” indicates that the Ab could be detected but cannot be purified easily; “NA” is not available.

**Figure 4:**
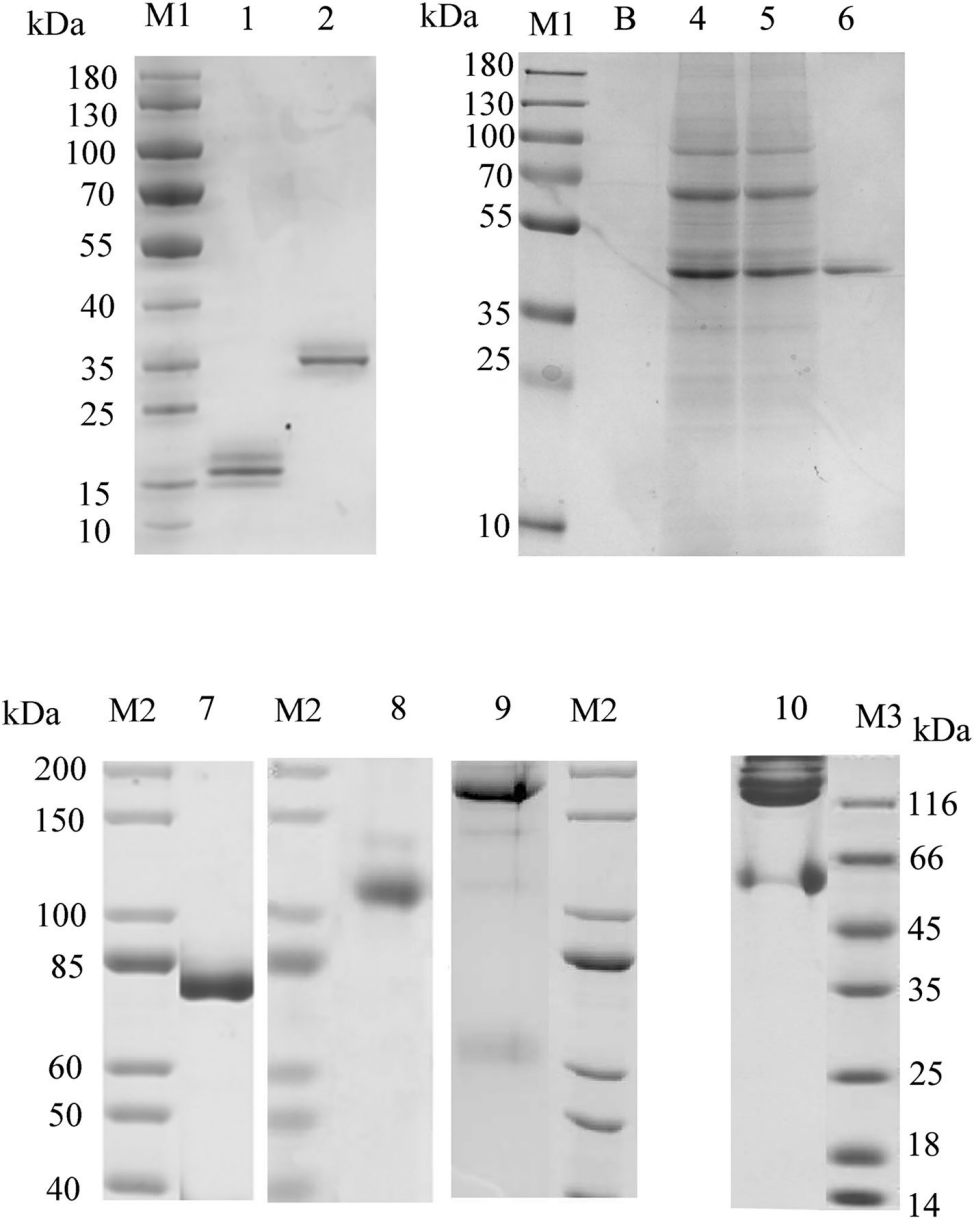
The SDS-PAGE gel of multiple VHH-B11 derived antibodies. The SDS–PAGE gel of multiple VHH-B11 derived antibodies. M1/M2/M3: the protein markers (Fermentas, USA). Lane 1: The VHH-B11 expressed by *P. pastoris* X33 (*pPICZα*); lane 2: The bivalent VHH-(G4S)3-VHH expressed by *Pichia pastoris* X33 (*pPICZα*); lane B: blank control; lane 4: the HEK293F culture medium superposition of the trivalent antibody VHH-(G4S)3-VHH-(G4S)3-VHH; lane 5: the Ni^+^ affinity column flow-through of the trivalent antibody VHH-(G4S)3-VHH-(G4S)3-VHH; lane 6: the purified trivalent antibody VHH-(G4S)3-VHH-(G4S)3-VHH; lane7: Llama VHH-human IgG4 hinge linker-IgG4 Fc chimeric HcAb (VHH-Fc); lane 8: the tetravalent VHH-llama long linker-VHH-human IgG4 hinge linker-IgG4 Fc (VHH-VHH-Fc); lane 9: the tetravalent VHH-(G4S)3-VHH-human CH1-IgG4 hinge linker- IgG4 Fc chimeric Ab (VHH-VHH-CH1-Fc); lane 10: the tetravalent VHH-human IgG4 hinge linker-IgG4 Fc-llama long linker-VHH chimeric Ab (VHH-Fc-VHH).

The principle of the first strategy is simple. Tandem VHHs were expressed in procaryotic or eukaryotic expression systems. The protein bands in lanes 1, 2, and 6 illustrated the intercomparison among the molecular weights (Fig. 4: lane 1, VHH: 17 kDa; lane 2, VHH-VHH: 35 kDa; lane 6, VHH-VHH-VHH: 51 kDa), and the medium supernatants and flow-through components are shown in lanes 4 and 5. The affinities between VHH-VHH (Fig. 2C) or VHH-VHH-VHH and PCSK9 are ~4 or 100-fold than that of VHH-B11 (Table 1).

The second type of IgG-like Abs mediate the augmentation of the biological half-life period because of Fc fusion that is expressed in the eukaryotic expression system, including *P. pastoris* and HEK293F cells. Five configurations were designed (Fig. 3F–J). Herein, VHH-Fc (Figs. 3F, 4, S6 lane 7: 75 kDa) has the hinge linker of the human IgG4 type, and the affinity increased ten-fold (VHH-Fc vs. VHH) (Fig. 2B, Table 1). VHH-CH1-Fc (Fig. 3G) is a recombinant heavy chain and has a very low expression due to lack of stability.

Next, three tetravalent Abs were designed. VHH-VHH-Fc (Figs. 3H, 4, S7 lane 8: ~ 110 kDa) has two hinge linkers; the llama long hinge peptide of the IgG2 type was used to link bivalent VHHs, and the hinge linker of the human IgG4 type was used to link human Fc. The Fc region of this tetravalent conformation is fully exposed. Also, its affinity increased ten-fold (VHH-VHH-Fc vs. VHH) (Fig. 2D, Table 1). As shown in Fig. 3I, VHH-VHH-CH1-Fc configuration has a low expression, and the protein band is ~ 155 kDa (Figs. 4, S8: lane 9). The last tetravalent Ab is VHH-Fc-VHH configuration (Figs. 3J, 4, S9: lane 10). The Fc region of this conformation acts as a linker. Similarly, its affinity to PCSK9 also increased ten-fold. A previous study reported an increased functionality of Fc-fused configuration^10^. Hence, the tetravalent Ab format VHH-VHH-Fc is relatively the best conformation based on the appropriate molecular weight, increased affinity, Fc exposure and medium expression level.

### Humanization and identification of key amino acids

**Figure 5:**
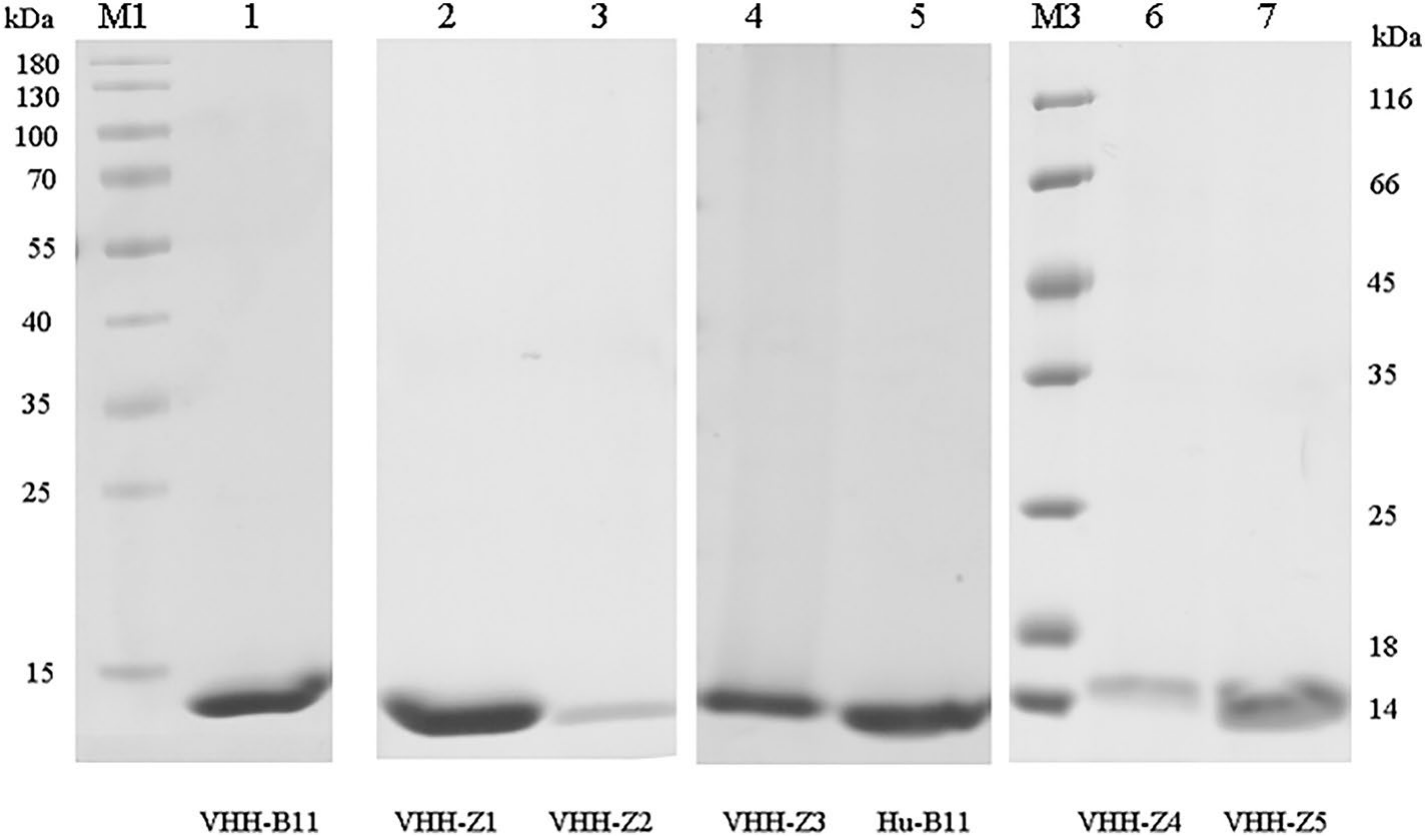
The SDS-PAGE gel of the VHH-B11 humanized antibodies. The SDS–PAGE gel of multiple VHH-B11 humanized antibodies expressed by *P. pastoris* X33. M1/M3: the protein markers (Fermentas, USA). Lane 1: Wild type VHH-B11; lane 2: VHH-Z1; lane 3: VHH-Z2; lane 4: VHH-Z3; lane 5: Hu-B11; lane 6: VHH-Z4; lane 7: VHH-Z5.

According to previous reports^16^, we summarized 17 hot spot amino acids of humanization in the three framework regions (FR 1–3) of VHH. To improve the degree of humanization of VHH-B11 (Fig. 5: lane1, we designed humanized VHH-B11 (Hu-B11) with all the 17 humanized amino acid mutation sites (Table 2). Surprisingly, Hu-B11 (Fig. 5 and Fig. S12: lane5) has a normal expression level similar to the wild-type VHH-B11 (~ 20 mg/L), but the affinity decreased by nine-fold such that Hu-B11 has an affinity interaction with hPCSK9 (4.4 nM vs. 39.7 nM) (Fig. 2A vs. Fig. S14A). However, each FR region has five or six humanization sites, and Hu-B11 has a high humanized degree; thus, affinity maturation is needed in the future. If not, it is highly likely that unsatisfactory pharmacodynamic evaluation effect will be obtained.

**Table 2:**
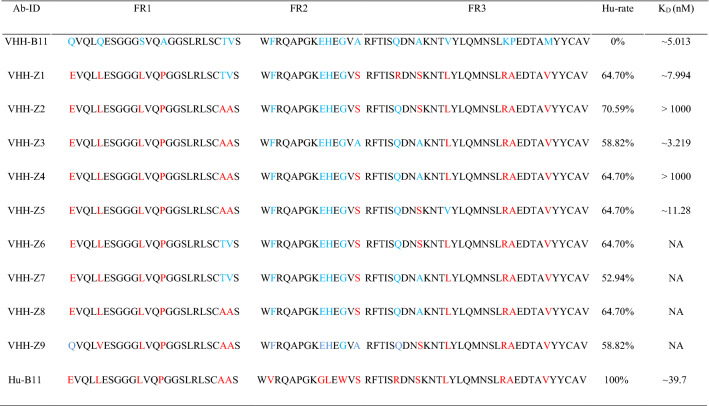
VHH-B11 Ab and its humanized mutants.

Ab ID is the name of VHH-B11 and each mutant. VHH-B11 is the wild type. The blue letters represent the locations of 17 “hotspot” humanization amino acids. Z1–Z9 are nine VHH-B11 humanized mutants, and each mutates at several of the hotspots. The mutated amino acids are labeled in red. Hu-B11 is a highly humanized mutant that mutates at all 17 hotspots. FR1-3 represents the three frameworks of VHH-B11. Hu-rate is short for the relative humanized rate (%). In this study, 17 amino acid mutations indicate 100% humanization rate. The Hu-rate is equal to the number of mutated amino acids divided by 17. K_D_(M) is the affinity between VHH-B11 mutant and hPCSK9 and is measured by the SPR technology at 25 ℃. “NA” represents not available.

In order to identify which amino acid position plays a critical role, we designed and expressed another nine VHH-B11 humanized mutants named by VHH-Z1-Z9 in *P. pastoris* (~ 15 kDa, Fig. 5, Figs. S10–S13 and Table 2). Most of these differed from each other by only one amino acid. For instance, VHH-Z1 (Fig. 5 and Figs. S11: lane 2, S14C) and VHH-Z6, VHH-Z2 (Fig. 5 and Fig. S11: lane 3) and VHH-Z5 (Fig. 5 and Figs. S13: lane 7, S14D), and VHH-Z3 (Fig. 5 and Figs. S12: lane 4, S14B) and VHH-Z4 (Fig. 5 and Fig. S13: lane 6) all have only one amino acid difference between each other. Among these, Hu-B11, VHH-Z3 and VHH-Z5 are expressed normally (~ 20 mg/L) in yeast, but the others have very low expression levels (< 1 mg/L), and VHH-Z9 is not expressed. There is only a single amino acid difference that can cause high or low expression and affinity (Fig. 5, Figs. S10–S13 and Table 2), as well as failure. Also, it could be speculated that these amino acid positions play critical roles. For instance, compared to the normal expression of VHH-Z1 (80Pro) (Fig. 5 and Figs. S11: lane 2, S14C), one amino acid difference (80Pro vs. 80Arg) in FR3 region causes low expression of VHH-Z6 (80Arg). Another replacement of the 87Val in the FR3 region of VHH-Z5 (normal expression, Fig. 5 and Figs. S13: lane 7, S14D) also led to a low expression and affinity of VHH-Z2 (87Lys). Similarly, one amino acid difference in FR2 region led to normal expression and no decline in the affinity of VHH-Z3 (54Ala) (Fig. 5 and Figs. S12: lane 4, S14B) but the very low expression and affinity of VHH-Z4 (54Ser). This finding suggested that these amino acid positions (no. 54, no. 80, and no. 87) may be crucial to the humanization of VHH-B11 protein.

On the other hand, the first five amino acids, “QVQLQ” of VHH-B11 were not indispensable because the protein expression, structure, and avidity of VHH-B11 were not affected without these initial amino acids (data not shown). In this case, VHH-Z9 expression failure could be attributed to the replacement of 83Ser in the FR3 region of VHH-Z3 (83Ala, normal expression, ~ 20 mg/L), indicating that 83Ala may also affect the normal formation of VHH-B11 Ab structure. When disregarding the initial five amino acids “QVQLQ”, VHH-Z2 and VHH-Z9 have only one amino acid difference at no. 54 (Ser vs. Ala). Among these, VHH-Z2 (Fig. 5 and Fig. S11: lane 3) has some expression, and Z9 has no expression, which further emphasizes the importance of no. 54 amino acid position on protein expression. It is worth noting that due to a poor fit, only three concentration gradients were showed the kinetic binding of VHH-Z1 and Z5 to the hPCSK9. (Fig. S14C,D). As for other humanized VHHs, they have poor or even no binding to PCSK9, so they are not shown.

### Epitope speculation and determination

In a previous study, we conducted SPR and sandwich ELISA (enzyme linked immunosorbent assay) assays and confirmed that the various PCSK9-binding epitope on VHH-B11 has different responses against Evolocumab^7^. However, the specific interaction epitope is yet unclear. To determine the interaction sites, we employed a random peptide library, phage display, and sequencing technology and characterized the amino acid of the binding epitope^17,18^.

**Figure 6:**
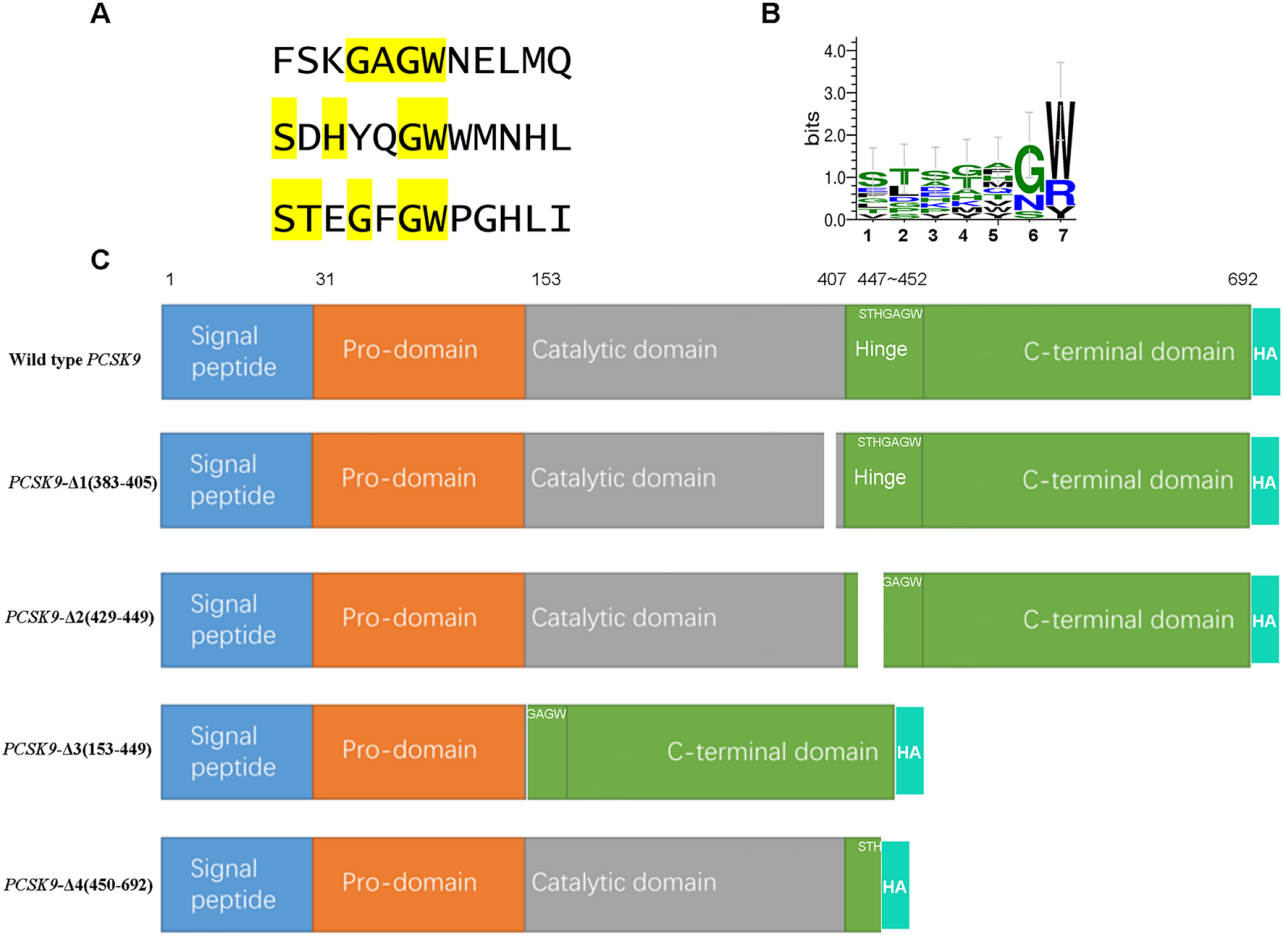
Epitope speculation and PCSK9 deletants design. (**A**) Epitope speculation. Many polypeptides with similar “GW” motif characteristics (for example*,* × S × GW, × T × GW, or × H × GW. × represents any amino acid) were identified from the random library (Table S2). Three peptides were listed as an illustration. (**B**) PCSK9 deletants design. Placing “GW” in the same amino acid position facilitated the identification of the possible common features (“STSGAGW”) of these short peptides after input in the Table S2 peptides by Weblogo 3 analysis tool. (**C**) Wild-type hPCSK9 and four hPCSK9-deletants lacked some modules: PCSK9-Δ1 (383–405), PCSK9-Δ2 (429–449), PCSK9-Δ3 (153–449), and PCSK9-Δ4 (450–692). hPCSK9 is composed of the signal peptide (amino acids 1–30), pro-domain (31–152), catalytic domain (153–407), hinge region (408–452), and C-terminal region (453–692). HA tag was fused for purification easily.

Sequencing revealed 321 Ab-binding peptides from groups A and B (Table S1). Further analyses showed similar motif characteristics (× S × GW, × T × GW or × H × GW) among several peptides; “ × ” represents one or more amino acids (Table S2). Three peptides are listed in Fig. 6A. If “GW” or “G” is placed in the same amino acid position, Weblogo 3 analysis tool identified the most common features (“STSGAGW”) of these short peptides after input of the peptides listed in Table S2 (Fig. 6B). Interestingly, the hinge region of hPCSK9, 447–452 amino acids (STHGAGW) met this characteristic. To verify this speculation, we designed and expressed wild-type hPCSK9 and four hPCSK9-deletants lacking some modules: PCSK9-Δ1(383–405), PCSK9-Δ2(429–449), PCSK9-Δ3(153–449), and PCSK9-Δ4(450–692) in HEK293F (Fig. 6C). The numbers in parentheses represent the deletion range of amino acids. For instance, PCSK9-Δ1(383–405) represents a PCSK9 deletant lacking 383–405 amino acids (“SGTSQAAAHVAGIAAMMLSAEPE”). The PCSK9-Δ1 deletant was regarded as the irrelevant contrast, and the other three deletants were experimental groups that the predicted epitope was broken by removing different modules.

**Figure 7:**
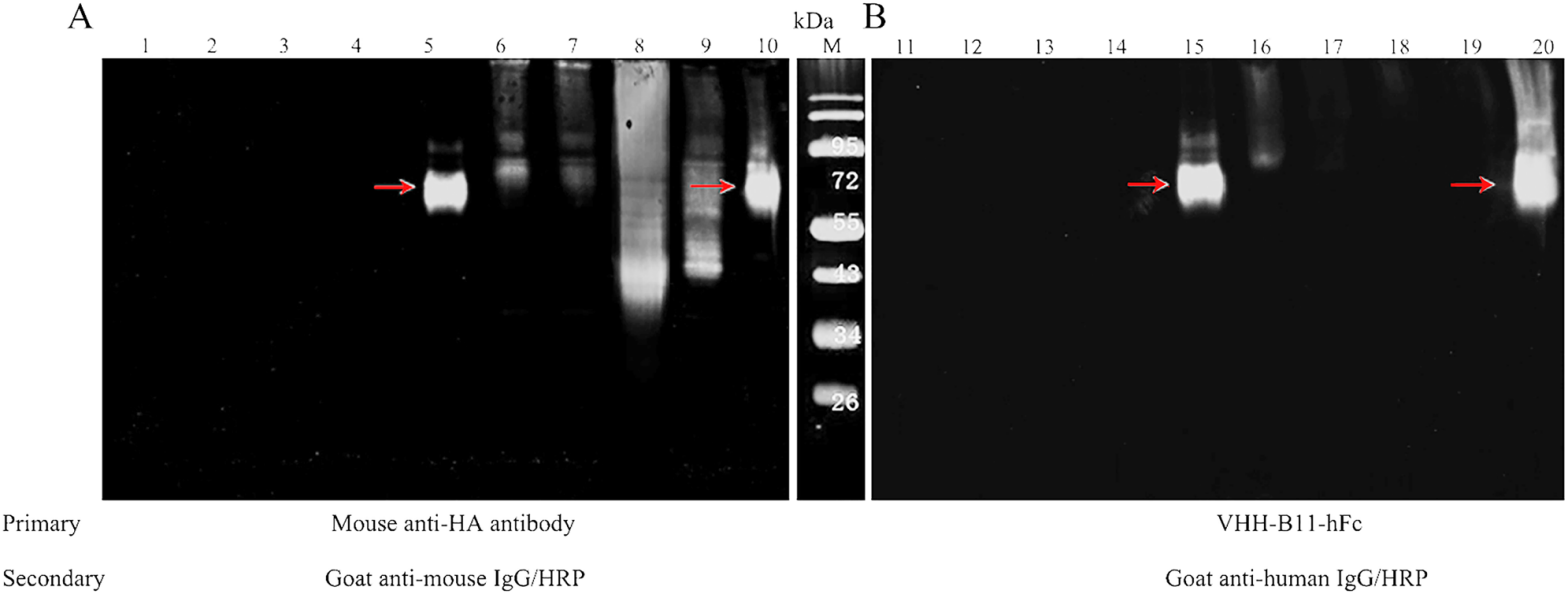
Immunoblot assay and epitope verification. Immunoblot assay was performed to verify the predicted epitope between PCSK9 and VHH-B11-Fc. PCSK9 and its deletants were loaded in the following sequence in groups of five samples (lanes 1–5 and 11–15: supernatants; lanes 6–10 and 16–20: cell precipitation lysate): PCSK9-Δ1 (383–405), PCSK9-Δ2 (429–449), PCSK9-Δ3 (153–449), PCSK9-Δ4 (450–692), and wild-type PCSK9 (red arrow). (**A**) The anti-HA Ab was used as primary Ab to check whether PCSK9 was expressed intracellularly (lanes 1–5) and extracellularly (lanes 6–10). The HRP-tagged goat anti-mouse IgG was used as the secondary antibody. (**B**) The B11-Fc Ab was used as a primary Ab to check whether PCSK9 and the deletants could still interact specifically with B11-Fc (lanes11–20). The HRP-tagged goat anti-human IgG was used as the secondary antibody.

An immunoblot assay was performed to verify this prediction. PCSK9 and its deletants were loaded in the following sequence in groups of five samples (supernatants and cell precipitation lysate): PCSK9-Δ1(383–405), PCSK9-Δ2(429–449), PCSK9-Δ3(153–449), PCSK9-Δ4(450–692), and wild-type PCSK9. As shown in Fig. 7A, anti-HA Ab was used as a primary Ab to assess whether PCSK9 was expressed intracellularly (Fig. 7A, lanes 1–5, supernatants) and extracellularly (Fig. 7A, lanes 6–10, cell lysate), while B11-Fc Ab was used as primary Ab to verify whether PCSK9 and the deletants could self-interact (Fig. 7B, lanes 11–15, supernatants and Fig. 7B, lanes 16–20, cell lysate). Anti-HA primary Ab group showed that wild-type PCSK9 was expressed extracellularly as well as intracellularly (~ 66 kDa & ~ 72 kDa, Fig. 7A, lanes 5 and 10), while all the PCSK9 deletants expressed only intracellularly (Fig. 7A, lanes 6–9). Furthermore, B11-Fc primary Ab group showed that wild-type PCSK9 (Fig. 7B, lanes 15 and 20) and the PCSK9-Δ1 deletant (the irrelevant contrast, Fig. 7B, lane16) could still be detected by B11-Fc because of their intact epitope, but the other three deletants (PCSK9 Δ2-Δ4, Fig. 7B lanes 17–19) not. These findings suggested that the broken epitope “STHGAGW” (no. 447–452) of the PCSK9 Δ2-Δ4 deletants would lead to the failure of detection by the specific Ab B11-Fc, deeming that this linear epitope is the interacting site between B11-Fc and PCSK9.

## Discussion

The single-domain Ab of camelidae is fragmentary and a therapeutic Ab. Due to the small size (~ 15 kDa) and the fast metabolism (~ 30 min) in the organisms^19–21^, appropriate modifications are essential to improve the pharmacokinetic and pharmacodynamic performances of single domain Abs^22,23^ that can be executed via VHH tandem (for example Cablivi: VHH-AlaAlaAla-VHH)^11^. However, human IgG Fc fusion is the general strategy for therapeutic VHH Abs^13,24^. For instance, KN035 Ab has VHH-Fc structure approved as an orphan drug by the US FDA in 2020^12^. Nevertheless, compared to conventional Abs, VHH tandem and/or Fc fusion is not optimal as it inevitably results in larger molecular weight, which in turn might alter the molecular structure and affect expression level and stability. Notably, human CH1 addition might also alter the structure of the camelid heavy chain Ab. For instance, VHH-Fc has a normal expression level, but VHH-CH1-Fc has a low expression level under the same conditions. Another example is VHH-VHH-Fc, with a normal expression level, but the quadrivalent VHH-VHH-CH1-Fc has a low expression level. These phenomena could be attributed to the lack of human CL1 domain coordination for CH1. Currently, researchers are seeking to maintain a balance of molecular weight, stability, pharmacodynamic efficacy and expression difficulty of the therapeutic Abs.

Ab humanization is crucial to reduce the immunogenicity of antigen (Ag)^25^. Low immunogenicity can reduce the occurrence of side effects^26^. In a previous study, we reported that human IgG4 Fc was fused to the llama VHH to form chimeras^7^. In the present study, we carried out 17 “hot spot” amino acid mutations to make it similar to the variable region of the human Ab gene^16^. However, the effect of immunogenicity reduction is to be evaluated in future studies^27^.

Epitope identification in the interaction between Ag and Ab is conducive to clarifying the mechanism of efficacy of the Ab^28^. Because of its unique advantages, camelid VHH Ab utilizes the conformation or charge complementarity for selective Ag binding, which mimics the conserved intracellular protein–protein interaction^29^. This characteristic is rarely exhibited by conventional IgG Abs. Reportedly, the interaction epitopes between the approved Evolocumab and PCSK9 are in the LDLR-binding site of the catalytic region^30^. In the present study, phage screening, random peptide library formation, and sequencing results proved that the VHH-B11-Fc binding epitope is in the hinge region of PCSK9. This phenomenon is rarely reported for anti-PCSK9 Ab. This method is economical, while the disadvantage is that only linear epitopes or linear parts of conformational epitopes can be identified. Thus, a better verification method, such as X-ray crystal diffraction, is required, especially for the conformational epitope. To obtain an ideal crystal of the Ag-Ab complex, some amino acid mutations on the molecular surface of protein^31^ are required; for example, the hinge region mutation, which in turn affects the binding of VHH-B11-Fc and PCSK9. Thus, we opted for the random peptide library approach.

In this study, we developed and characterized a camelid VHH-B11 Ab. The druggability of VHH-B11 has been enhanced adequately. Both VHH tandem and Fc fusion have been used to form polyvalent Ab, such as tetravalent VHH-VHH-Fc. It has a high affinity to hPCSK9 (0.1 nM). Moreover, it has a relatively high expression level (> 10 mg/L). Combined with the identified epitopes and good humanization results, this study provides experimental evidence for improved druggability of camelid-derived VHH Ab.

## Methods

### Monovalent nanobody expressed in the *E. coli* system

VHH-B11 was expressed in three systems (*E. coli*, *P. pastoris* and HEK293F mammalian cells) to investigate the difference in the nanobodies. The BL21 *E. coli*, TG1 *E. coli*, and the *pET28a* vector were preserved in our laboratory. HB2151 *E. coli* and *pMECS* expression were obtained as a kind gift from the University of Brussels, Belgium^32^. After culture for 4–6 h in LB or TB medium (0.72 M KH_2_PO_4_, 170 mM K_2_HPO_4_, 1.2% peptone, 2.4% yeast extract, 0.4% glycerol, 100 μg/mL ampicillin, 0.1% glucose, and 2 mM MgCl_2_), 1 mM IPTG was added when OD_600_ was 0.8–1.0 to induce VHH expression overnight. The cell pellet was collected by centrifugation at 10,000 rpm for 10 min. Ultrasonication was used to release the intracellular expression (BL21 *E. coli*). The TES buffer (0.2 M Tris–HCl pH 8.0, 0.5 mM EDTA, 0.5 M sucrose) was used for TG1 and HB2151 bacterial lysis^32^. The solvent was replaced with phosphate buffer for the immunoaffinity purification using ultrafiltration (3 kDa, Millipore, USA) for good purification results. The specific experimental procedures were carried out according to the manufacturer’s instructions.

### Ab expression in *P. pastoris* system

In this study, *P. pastoris* X33 and *pPICZα* plasmids (Life Invitrogen, USA) were used to assess the secreted level of monovalent and partial multivalent VHH Abs, which improves the expression efficiency and facilitates purification without cell lysis. The recombinant *pPICZα-VHH* vectors were linearized by single restriction endonuclease cleavage by *SalI* and purified by agarose gel electrophoresis. Subsequently, after electroporation, the bacterial suspension was spread on YPD solid medium plate (1% yeast extract, 2% peptone, 2% dextrose glucose, 2% agar). Then, zeocin was added in gradient (100, 200, 400, and 800 mg/mL) to the YPD medium for screening the stable expression of yeast strains. BMGY medium (1% yeast extract, 2% peptone, 3 g/L K_2_HPO_4_, 11.8 g/L KH_2_PO_4_, 0.02% biotin, 1% glycerol, and 1 × yeast nitrogen base source medium) was used to expand and propagate *P. pastoris* for 3 days*.* Methanol was added every 24 h to induce the Ab expression in the new BMMY medium (1% yeast extract, 2% peptone, 3 g/L K_2_HPO_4_, 11.8 g/L KH_2_PO_4_, 0.02% biotin, 0.5% methanol, and 1 × yeast nitrogen base source medium). After 5 days of inducing expression, the cell supernatant was collected by centrifugation at 12,000 rpm for 10 min. If the volume was large (> 2 L), ammonium sulfate precipitation method is recommended to precipitate the protein. For adequate immunoaffinity purification results, the protein precipitate needs to be solubilized in the equilibration buffer (such as phosphate buffer).

### Ab expression in HEK293F mammalian cell system

To express Abs with advanced and complex structures, HEK293F mammalian cell (Sinobiological, China) expression system was selected for VHH-derived Ab secretory production. For easy purification, a special serum-free medium (SMM 293-TII, Sinobiological, China) was used to support the growth and transfection of HEK293F cells. The *pCDNA3.4* (purchased from Life Invitrogen) was used for the transient transfection (Sinofection, Sinobiological). Briefly, the freshly passaged cells were seeded at a density of 0.5 × 10^6^/mL and incubated at 37 °C (175 rpm, 5% CO_2_) overnight. At the time of transfection, the suspension cells were in the log growth phase. Next, the endotoxin-free plasmid *pCDNA3.4* was diluted with 0.15 M NaCl solution at 20 μg/mL (optimization recommended), and the transfection reagent (DNA: reagent = 1: 4) was diluted in the same volume. Then, the diluted DNA was combined with the diluted transfection reagent, incubated for 20 min at room temperature, and added drop-wise to the cells, followed by gentle shaking to ensure even distribution. Then, the cells were incubated at 37 °C in a CO_2_ shaker (175 rpm) for 48 h, and then the cell culture supplement (3.5%) was added every 48 h.

After 5 days of shaking cultivation, the cell supernatant was collected by centrifugation at 12,000 rpm for 10 min. The specific experimental procedures were carried out according to the manufacturer’s instructions.

### Purification

After culture and expression, the AKTA pure25 (GE Healthcare, USA) was used for automated purification after setting up the program. The 5 mL Histrap FF purification column (GE Healthcare) was used for His-tag VHH protein purification. The 5 mL Protein A purification column (GE Healthcare) was used to purify the Fc-fused Abs. After purification, the solvent of the protein solution should be replaced with the phosphate buffer in the 15-mL ultrafiltration tube (Millipore, USA). The specific experimental procedures were according to the manufacturer’s instructions.

### SPR assay

SPR technology was adopted to determinate the affinities between the VHH-based Abs and hPCSK9 antigen. First, recombinant hPCSK9 (his-tagged, Sinobiological, China) was diluted to 10 μg/mL with 10 mM sodium acetate (pH = 4.0). Then hPCSK9 protein was captured on the flow cell 2 of the CM5 chip (GE Healthcare, USA, Cat# 29104988) at 790 response unit (RU), with flow cell 1 as the blank. All the SPR assays were executed under 25 °C using the Biacore T200 (GE Healthcare, USA). The binding and dissociation time was set at 120 s and 300 s (or 350 s) respectively. A five (or three points), double fold dilution of different molar concentrations of these VHH-based proteins was injected and the sensorgrams were fitted with a floating Rmax using the built-in evaluation software of the Biacore T200. The glycine (pH = 2.3) was used as regeneration buffer. The affinity (K_D_, nM) is calculated as followings: K_D_ = k_off_/k_on_. k_off_ (1/s) is the dissociation constant and k_on_ (1/Ms) is the binding constant.

### Phage random peptide library screening

Ph.D.-12 phage random peptide library (New England Biolabs) sequencing technologies were utilized to identify the interaction peptides^18^. This method was based on expressing recombinant random peptides on the surface of the phage and selecting the potential interacting peptides bound to the solid phase. The initial experimental steps are similar to the phage-ELISA procedures^18,32,33^. Briefly, ELISA wells were coated with the VHH-B11-Fc Ab in duplicate at 4 ℃ overnight and blocked with 3% bovine serum albumin (BSA) after five washes with 1 × PBST, termed group A and group B, respectively. A no-coating well was set up as the control group. After blocking and washing, Ph.D.-12 phage random peptide library was added, and then the binding Ab-phage complex was reserved after five washes with 1 × PBST washing. With the complex washed off with triethylamine, the sequencing library was constructed from the DNA obtained by high-temperature lysis, as described previously^18^. Essentially, the DNA library was generated by two consecutive rounds of extension PCR using Q5 Hot Start High-Fidelity DNA Polymerase (New England Biolabs). After quality control, the barcoded DNA library was sequenced on Novaseq 6000 under the paired-end 2 × 150 sequencing (PE150) strategy.

### Data processing, analysis and epitope speculation

To acquire high-quality reads from raw data, Trimmomatic software was used to filter out the low-quality reads^34^. The 150-bp PE reads with the exact same insertion sequence were considered clean data for subsequent analysis. A pair of 8-bp barcodes and 36-bp variable DNA inserts were considered corresponding to the 12-amino acid polypeptide for single reads. This pair of 8-bp barcodes distinguished the VHH-B11-Fc Ab sample from the control. Next, the peptide number of the Ab was counted and normalized to the total peptide number. If one peptide appeared in our sample and not the control, the count was set as 1 in control. The number of these peptides was not calculated for the total control sample counts. In addition, the arithmetic mean of each peptide was calculated for the control and added into the matrix as the control column. The resulting matrix was defined as the normalized count matrix. Subsequently, the Ab-binding peptides were determined by comparing between the Ab and the control. Since no Ab was added to the control, all the peptides were considered noise. In order to identify the binding peptides for the downstream analyses, the cutoff value was determined as described previously. Only the eligible peptides were recognized as binding peptides, according to a previous report^18^.

### Epitope determination by Western blot

We designed four hPCSK9 deletants for epitope determination and disrupted (or removed) the putative epitopes of three to assess if the binding between the VHH-B11-Fc Ab and PCSK9 was affected^30^. For the remaining deletant, we removed an unrelated epitope as an irrelevant control to verify whether this affects the Ab-hPCSK9 interaction. Moreover, the wild-type hPCSK9 served as the positive control. Also, the HEK293F mammalian cell system was selected for hPCSK9 expression. hPCSK9 and all its deletants were expressed with HA tag. A volume of 10 μL medium supernatant and 1 μL of 1 mL cell precipitation lysate were used for Western blot. After non-reducing PAGE, the proteins were electrotransferred to a polyvinylidene difluoride membrane (PVDF; Millipore) at 400 mA for 70 min. The membranes were then blocked with 3% BSA and probed with two primary Abs (mouse anti-HA Ab or the VHH-B11-Fc Ab) at room temperature for 2 h, followed by two corresponding secondary Abs (horseradish peroxidase (HRP)-tagged goat anti-mouse IgG or HRP-tagged goat anti-human IgG) at 37 ℃ for 1 h to determine the PCSK9 protein levels. The immunorective bands were detected using the enhanced chemiluminescence system (Biouniquer, Nanjing, China). Herein, the mouse anti-HA primary Ab was used to evaluate whether the four PCSK9 deletants were expressed extracellularly or intracellularly. The VHH-B11-Fc primary Ab was used to check whether the binding between the Ab and the PCSK9 deletants was affected.

### Ethics approval and consent to participate

Permission for this study was granted by the Bioethics and Biological Safety Review Committee of BGI-Shenzhen (NO. FT 17064). We confirm that all experiments were conducted in accordance with relevant guidelines and regulations.

## Supplementary Information

Supplementary Figure 1.

Supplementary Figure 2.

Supplementary Figure 3.

Supplementary Figure 4.

Supplementary Figure 5.

Supplementary Figure 6.

Supplementary Figure 7.

Supplementary Figure 8.

Supplementary Figure 9.

Supplementary Figure 10.

Supplementary Figure 11.

Supplementary Figure 12.

Supplementary Figure 13.

Supplementary Figure 14.

Supplementary Information 15.

Supplementary Tables.

## Data Availability

The data that supports the findings of this study are available in the supplementary material of this article.

## Abbreviations

(G4S)3-linker: GlyGlyGlyGlySerGlyGlyGlyGlySerGlyGlyGlyGlySer
Antibody: Ab
Antigen: Ag
BSA: Bovine serum albumin
DNA: Deoxyribonucleic acid
* E. coli *: *Escherichia coli*
ELISA: Enzyme linked immunosorbent assay
Fc: The constant region fragment of the human immunoglobulin gamma
FR: Framework region
HcAb: Heavy chain antibody
HEK293F: Human embryonic kidney 293F cells
hPCSK9: Human proprotein convertase subtilisin/kexin type 9
HRP: Horseradish peroxidase
Hu-B11: Humanized VHH-B11
IgG: Immunoglobulin gamma
IPTG: Isopropyl-β-d-thiogalactoside
kDa: Kilo dalton
LDL: Low density lipoprotein
LDL-c: Low density lipoprotein cholesterol
LDLR: Low density lipoprotein receptor
OD_600_: The optical density value at 600 nm
PCSK9: Proprotein convertase subtilisin/kexin type 9
PCR: Polymerase chain reaction
*P. pastoris*: *Pichia pastoris*
RFU: The relative fluorescence unit
RU: Response units
SD rat: Sprague Dawley rat
sdAb: Single domain antibody
SPR: Surface plasmon resonance
TES buffer: 0.2 M Tris–HCl (pH 8.0), 0.5 mM EDTA, 0.5 M sucrose
VHH: High variable region of the heavy chain antibody
kDa: Kilo dalton
PBST: Phosphate buffer solution with 0.05% tween 20
PE: Pair end
Ph.D.-12 library: It is a combinatorial library of random 12-mer peptides fused to a minor coat protein (pIII) of M13 phage. The displayed peptide is expressed at the N-terminus of pIII.
RNA: Ribonucleic acid
YPD: 1% Yeast extract, 2% peptone, 2% dextrose glucose
