# Supplementary figures and images for: Development and characterization of a camelid derived antibody targeting a linear epitope in the hinge domain of human PCSK9 protein
Source: Sci Rep. 2022 Jul 16;12:12211. doi: 10.1038/s41598-022-16453-3 (PMC9288512; doi:10.1038/s41598-022-16453-3)

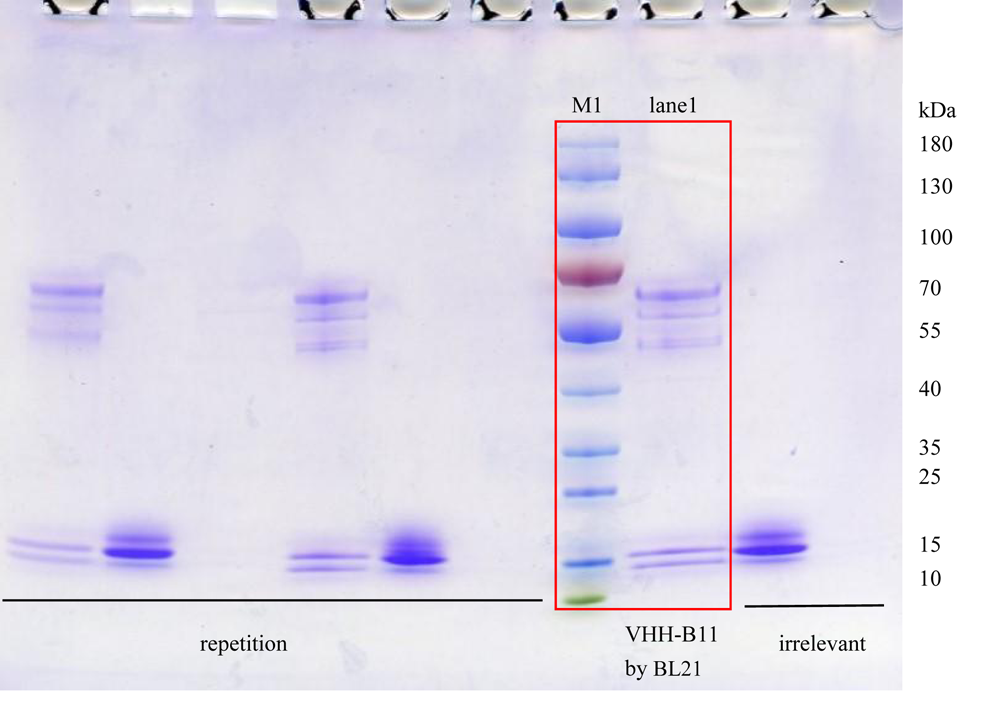

Supplement: Supplementary file 1 — Supplementary Figure 1. [file 41598_2022_16453_MOESM1_ESM.tif]

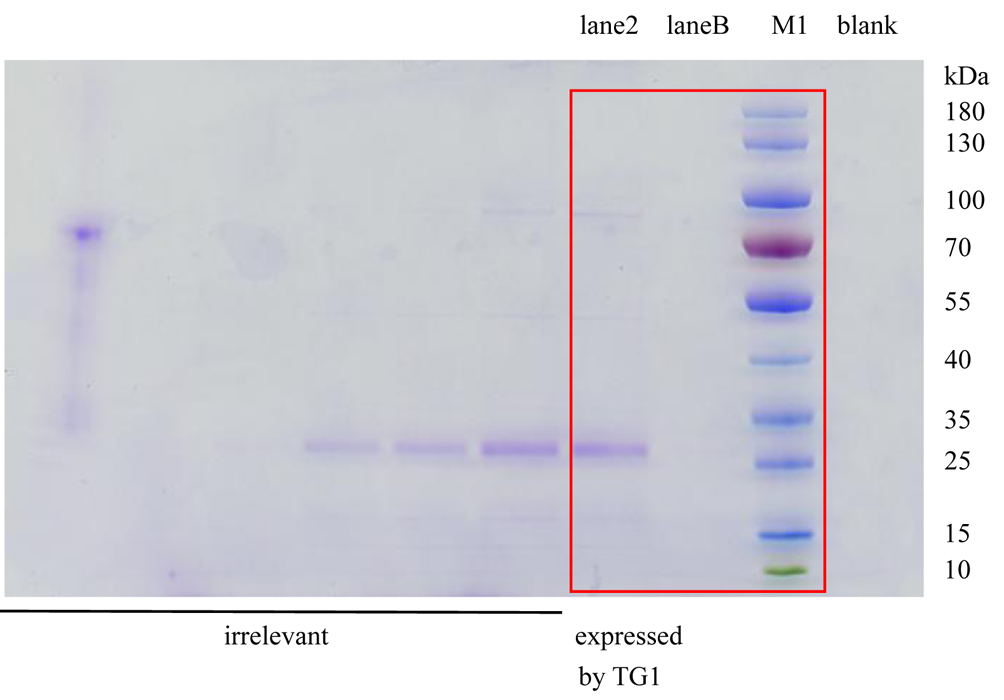

Supplement: Supplementary file 2 — Supplementary Figure 2. [file 41598_2022_16453_MOESM2_ESM.tif]

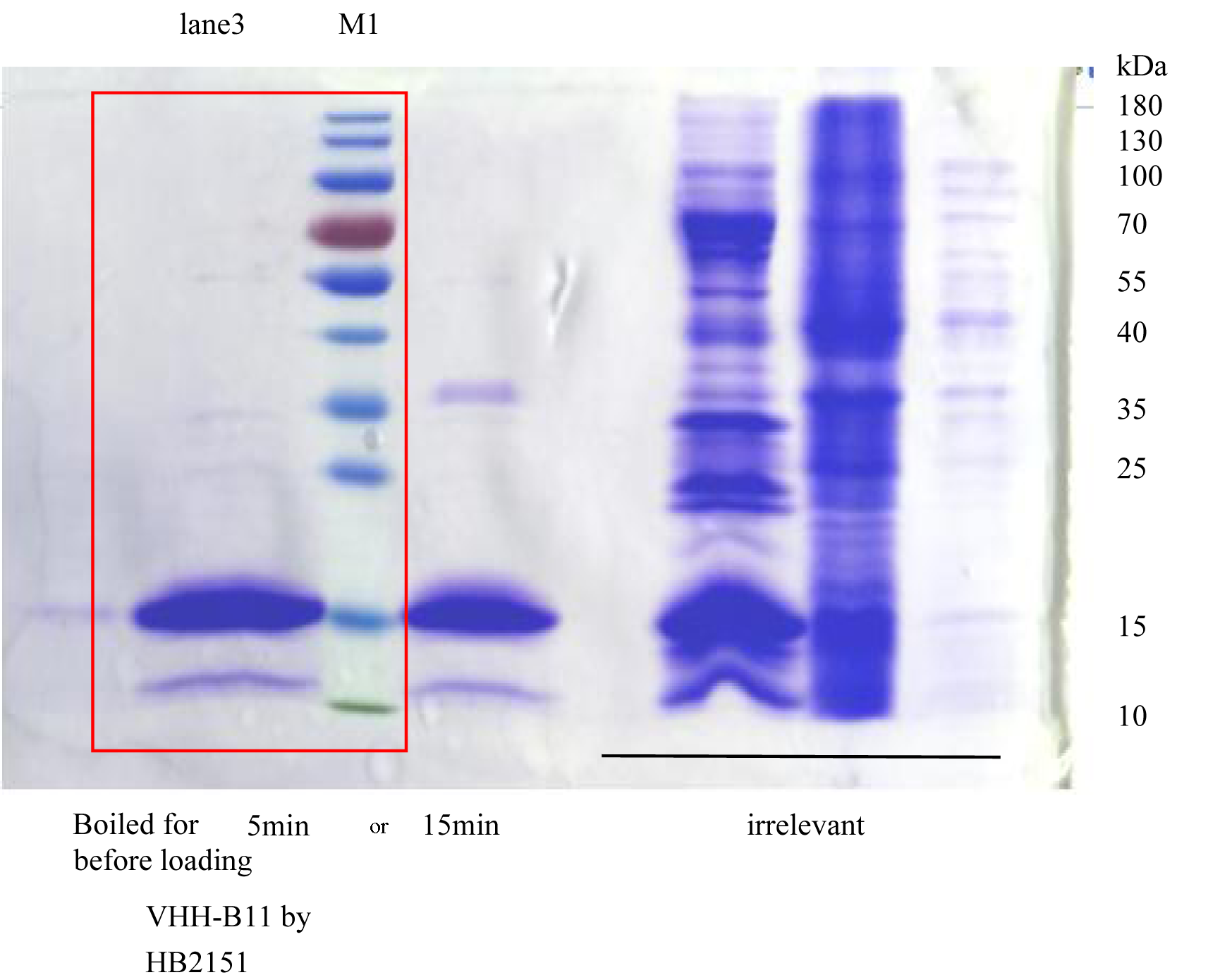

Supplement: Supplementary file 3 — Supplementary Figure 3. [file 41598_2022_16453_MOESM3_ESM.tif]

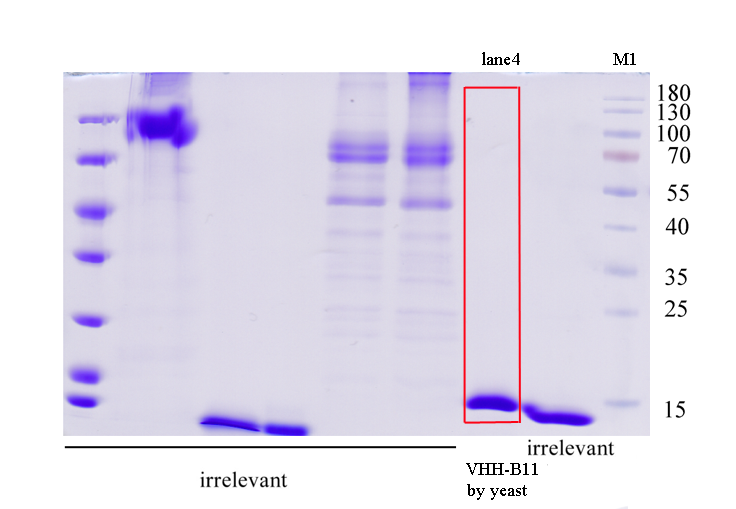

Supplement: Supplementary file 4 — Supplementary Figure 4. [file 41598_2022_16453_MOESM4_ESM.tif]

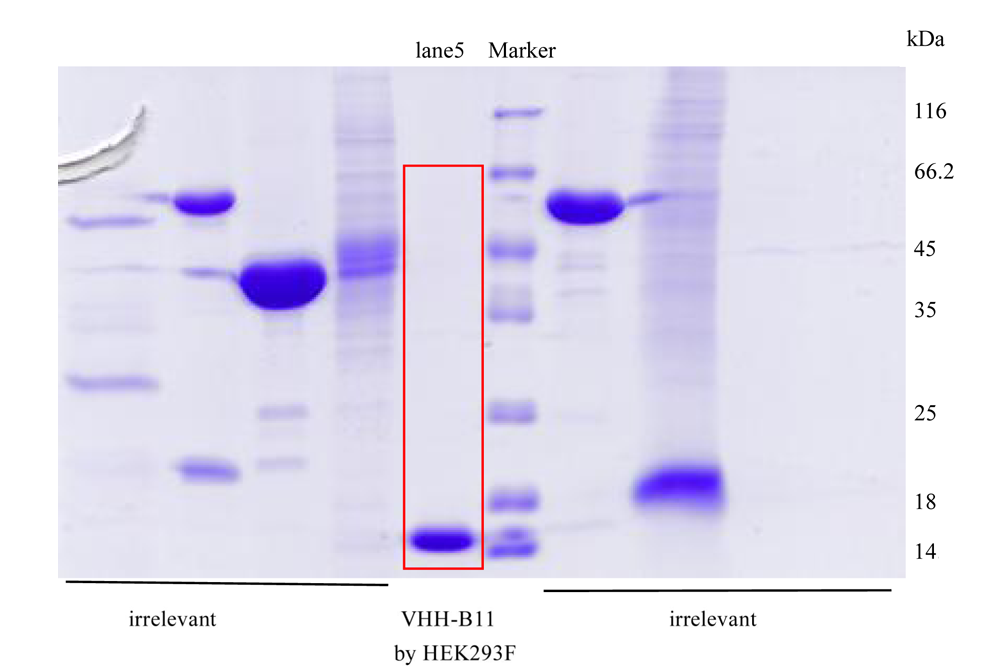

Supplement: Supplementary file 5 — Supplementary Figure 5. [file 41598_2022_16453_MOESM5_ESM.tif]

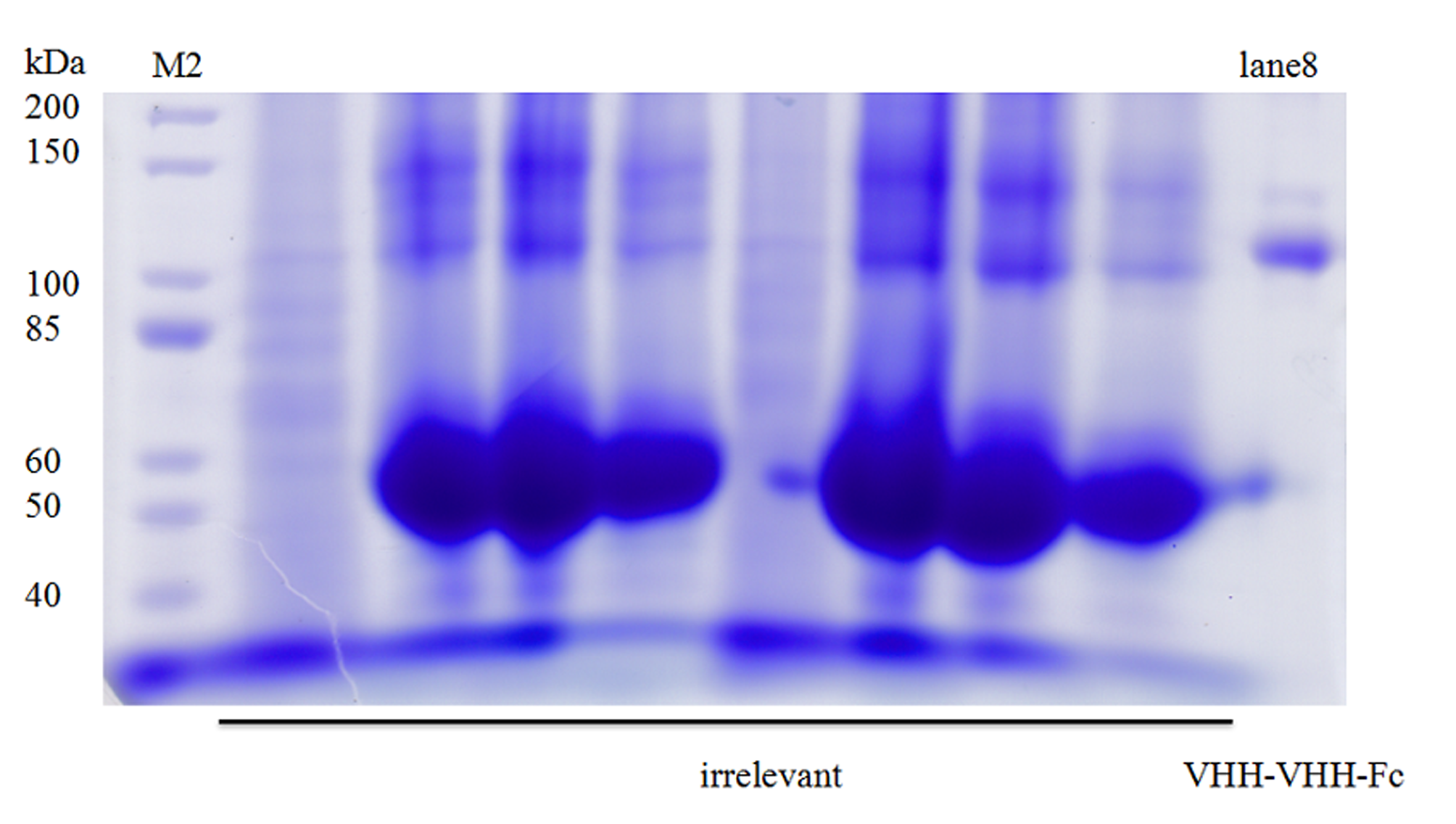

Supplement: Supplementary file 7 — Supplementary Figure 7. [file 41598_2022_16453_MOESM7_ESM.tif]

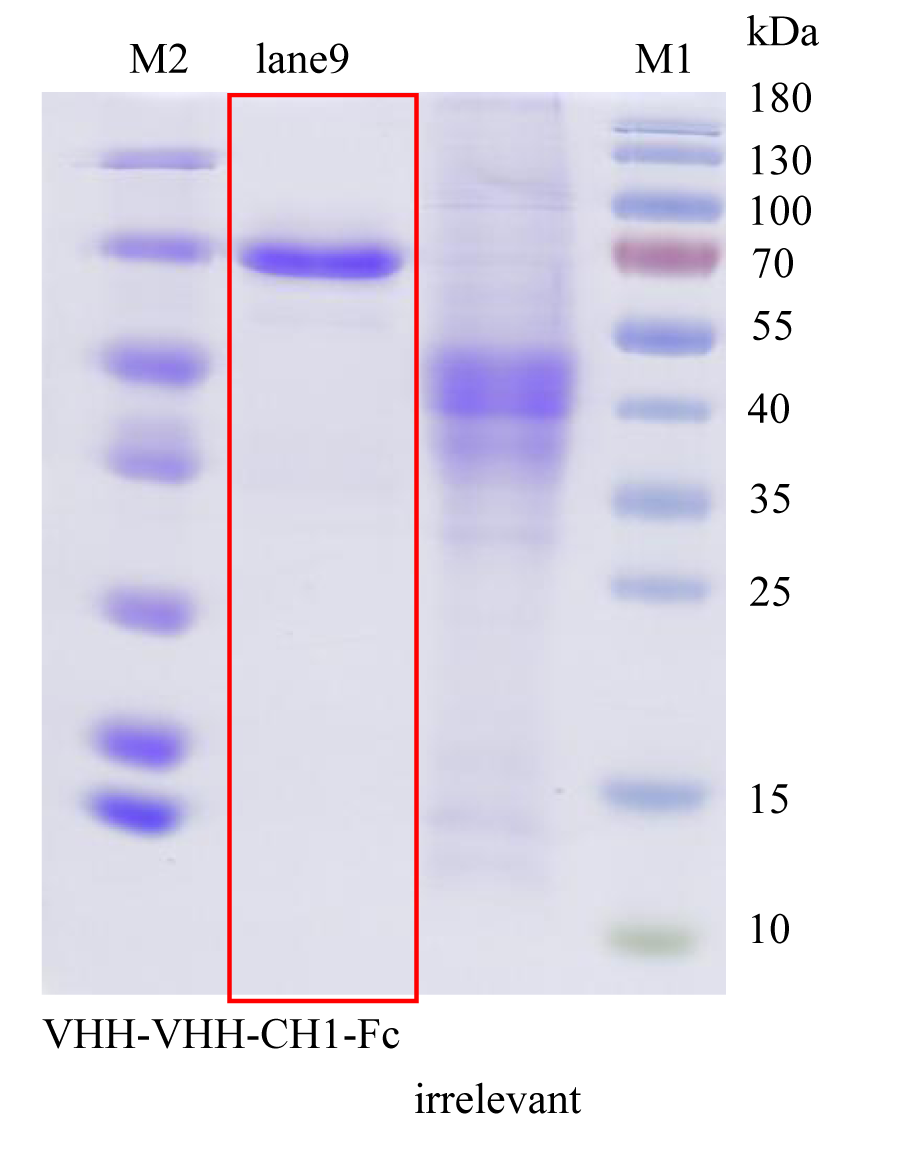

Supplement: Supplementary file 8 — Supplementary Figure 8. [file 41598_2022_16453_MOESM8_ESM.tif]

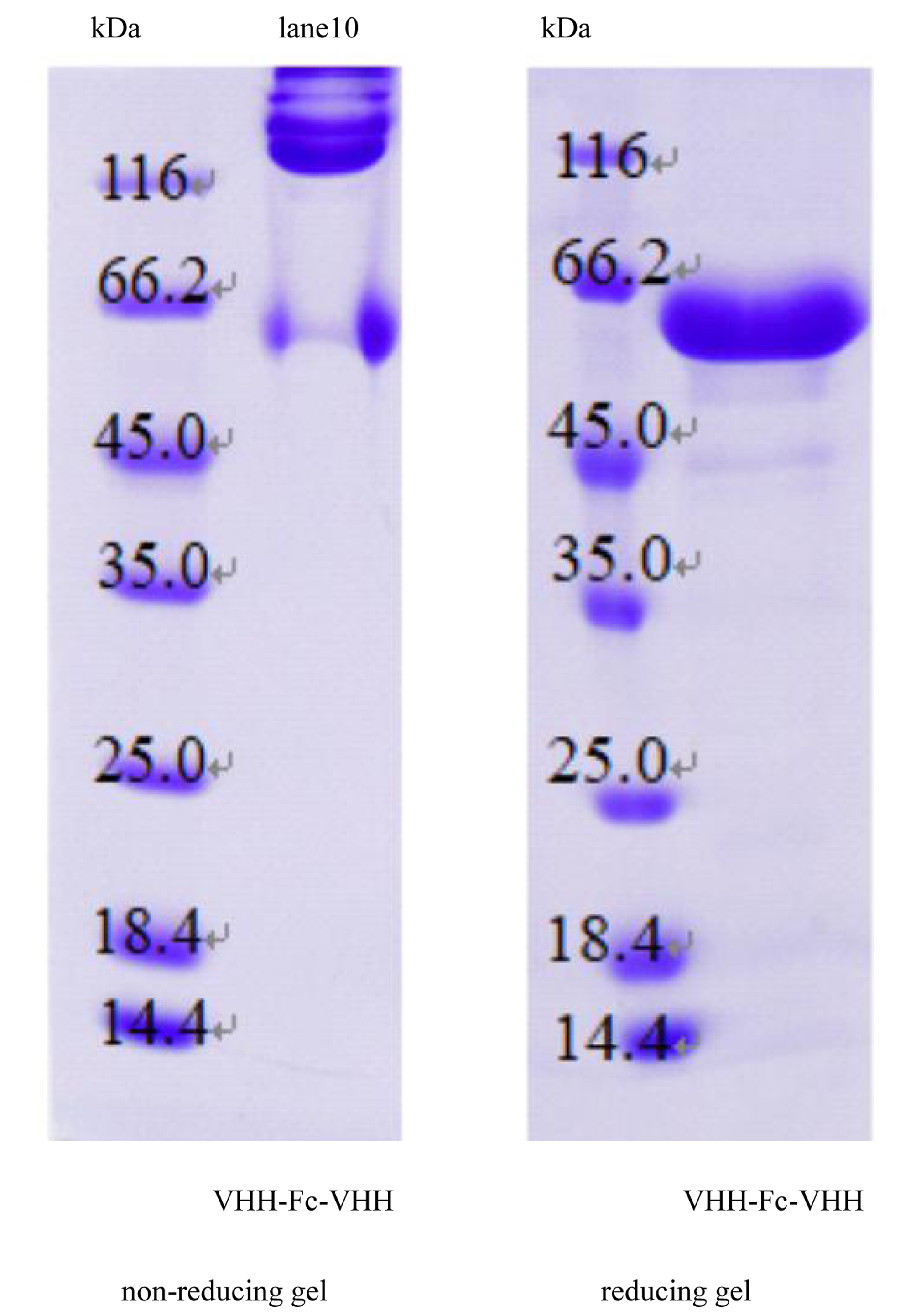

Supplement: Supplementary file 9 — Supplementary Figure 9. [file 41598_2022_16453_MOESM9_ESM.tif]

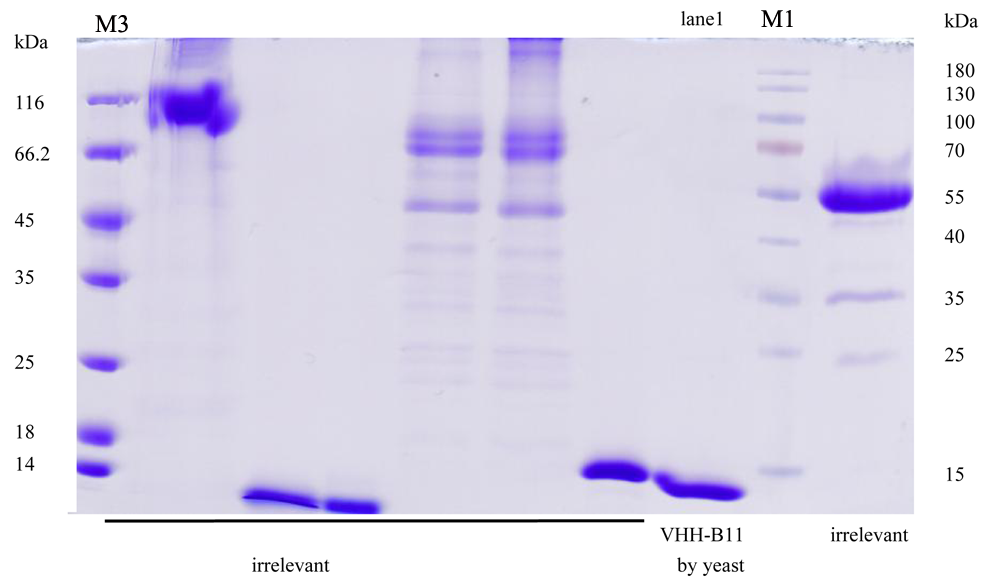

Supplement: Supplementary file 10 — Supplementary Figure 10. [file 41598_2022_16453_MOESM10_ESM.tif]

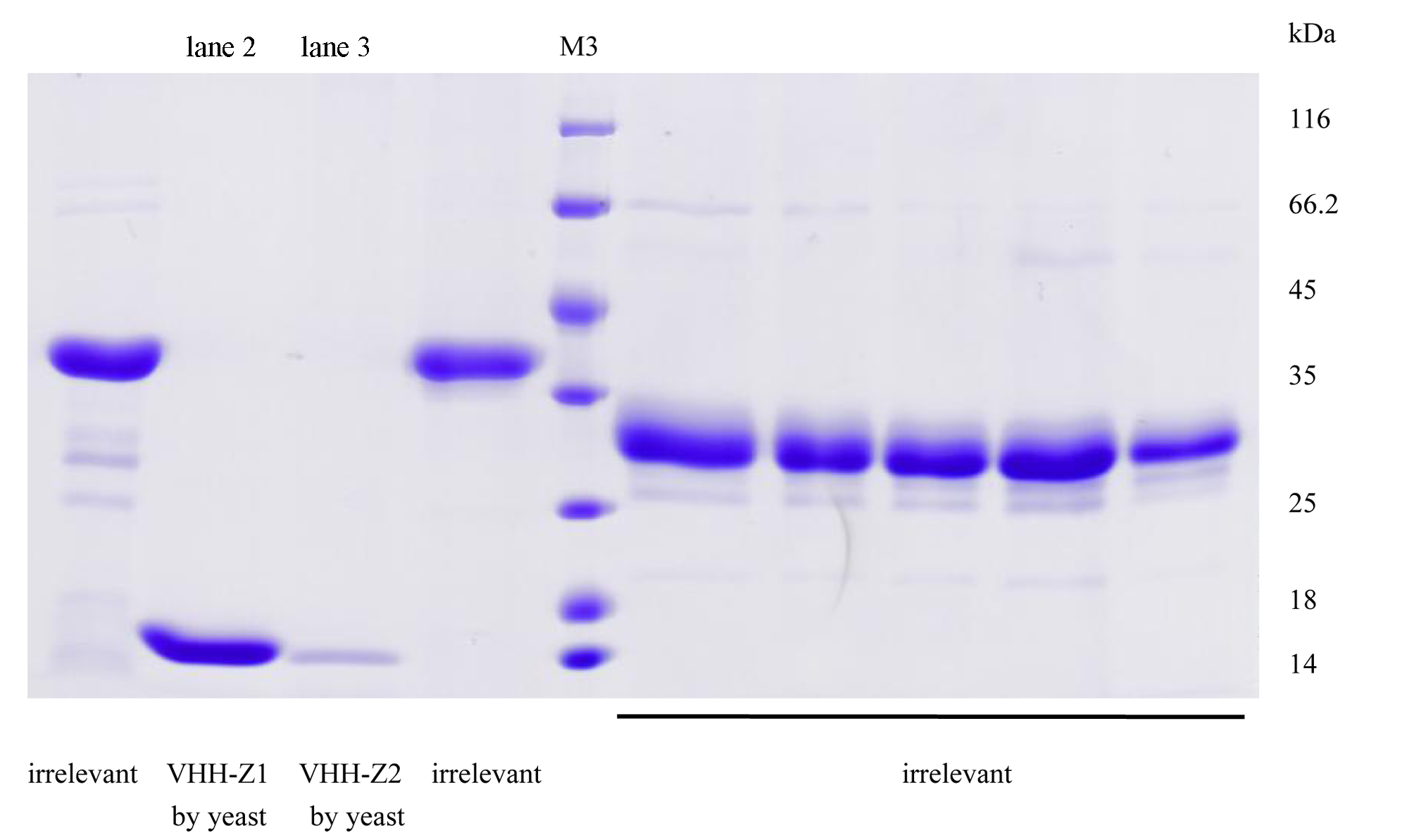

Supplement: Supplementary file 11 — Supplementary Figure 11. [file 41598_2022_16453_MOESM11_ESM.tif]

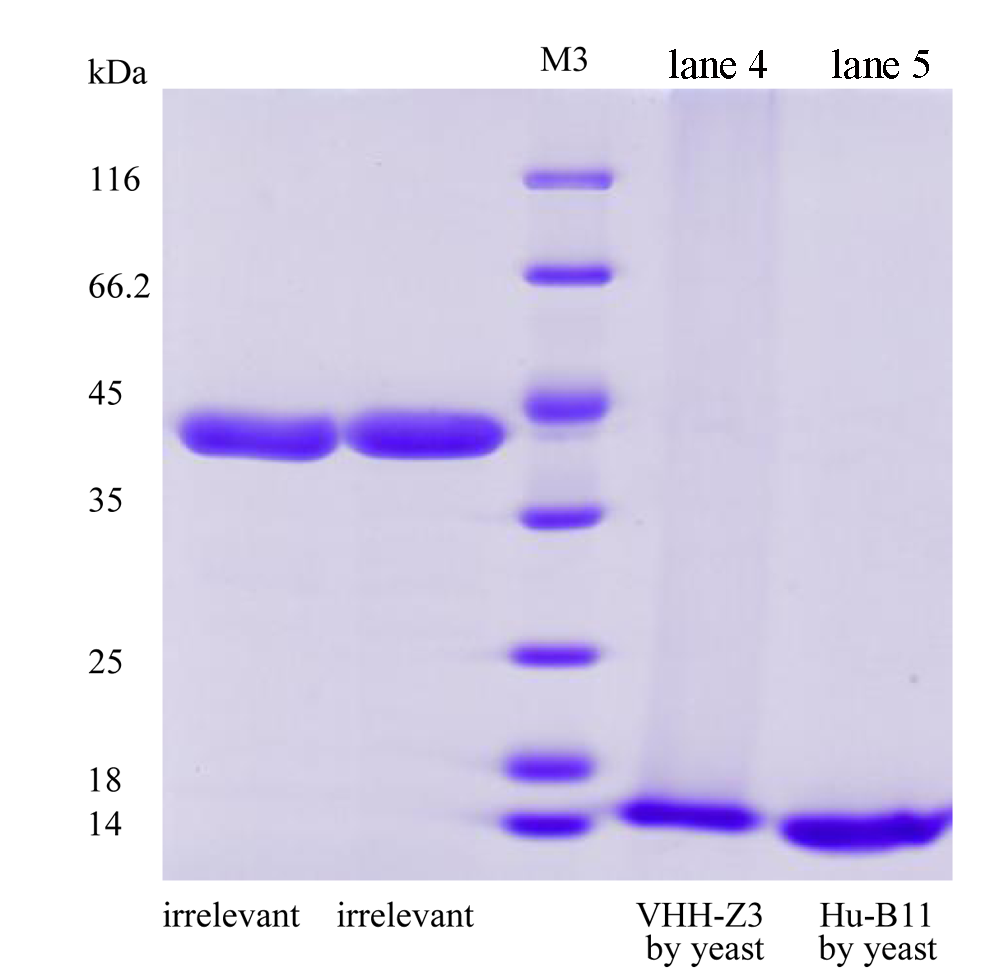

Supplement: Supplementary file 12 — Supplementary Figure 12. [file 41598_2022_16453_MOESM12_ESM.tif]

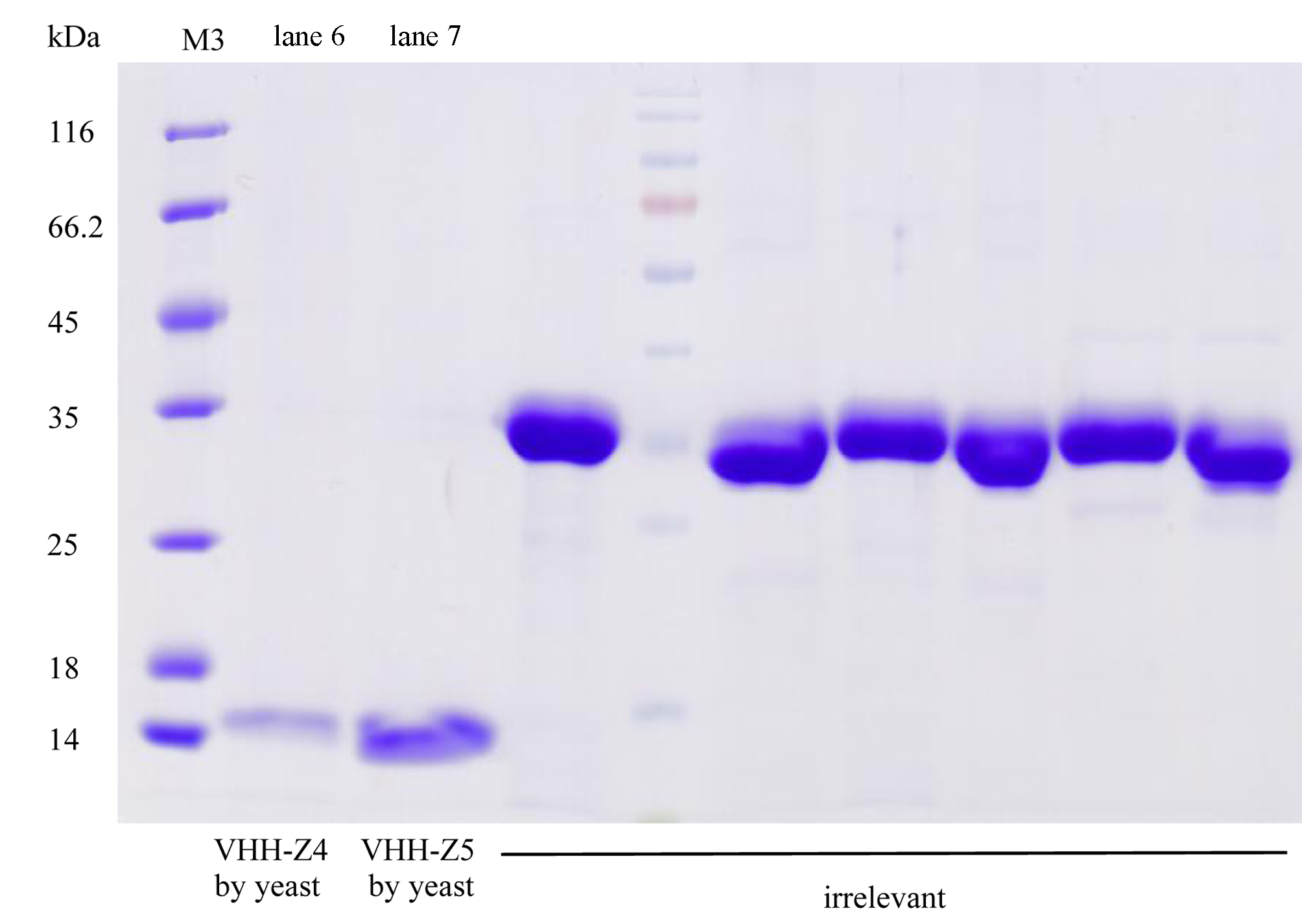

Supplement: Supplementary file 13 — Supplementary Figure 13. [file 41598_2022_16453_MOESM13_ESM.tif]

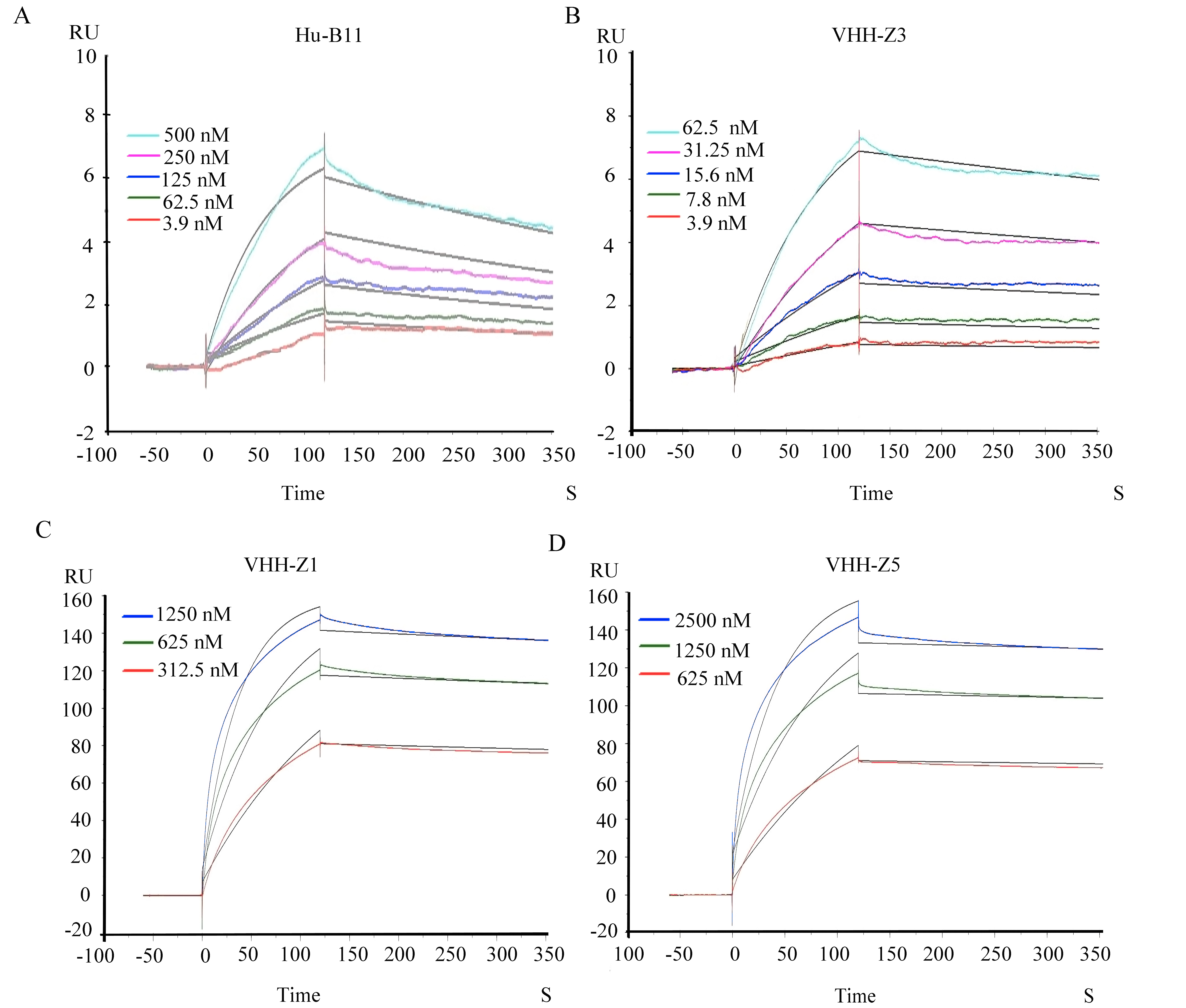

Supplement: Supplementary file 14 — Supplementary Figure 14. [file 41598_2022_16453_MOESM14_ESM.tif]
