# Supplementary material for: Development and characterization of a camelid derived antibody targeting a linear epitope in the hinge domain of human PCSK9 protein
Source: Sci Rep. 2022 Jul 16;12:12211. doi: 10.1038/s41598-022-16453-3 (PMC9288512; doi:10.1038/s41598-022-16453-3)
Supplement: Supplementary file 15 — Supplementary Information 15. [file 41598_2022_16453_MOESM15_ESM.docx]

**Fig. S1-S5 The original gel photos of the Fig. 1**

**Fig. S6-S9 The original gel photos of the Fig. 4**

**Fig. S10-S13 The original gel photos of the Fig. 5**

**Fig. S14 Affinity test between the VHH-B11 humanized Abs and human PCSK9**

The affinities were also determined by SPR technology. Two-fold dilution of the humanized Abs (five or three concentrations in nM) expressed by *P. pastoris* X33 were loaded to bind the hPCSK9 antigen coated on the CM5 chip. Each colored line represents a concentration (in nM) of the Abs. The black lines were the automatic fitting curves by the built-in evaluation software of Biacore T200. The horizontal axis represents the injection time, and the vertical axis represents the response unit (RU) value of the humanized Abs interaction with hPCSK9. The VHH injection time point was set as 0 s by the evaluation software. A. Hu-B11; B. VHH-Z3; C. VHH-Z1; D. VHH-Z5.

**Table S1 The Ab-binding peptides acquired from the phage screening and sequencing**

Note: There are total 321 Ab-binding peptides acquired from the phage screening and sequencing. Among them, 115 were identified in group A and 206 in group B.

**Table S2 Identified polypeptides with “GW” similar characteristics**

Note: A total of sixteen 12-amimo acids-peptides were identified with similar “GW” characteristics from groups A and B. These were listed after placing “GW” or “G” in the same location. Herein, both groups identified polypeptides “EGYHHGWIHMPS” and “VLSTTSRIGWWM” simultaneously. “Binding reaction” represents the orientation of the peptide-binding VHH-B11-Fc (i.e., forward binding or reverse binding). The forward binding is labeled as “F” and the reverse binding as “R”. The “×S×GW, ×T×GW, and ×H×GW” similar characteristics were labeled in yellow (forward binding) and red (reverse binding).
