# Supplementary material for: Development and characterization of a camelid derived antibody targeting a linear epitope in the hinge domain of human PCSK9 protein
Source: Sci Rep. 2022 Jul 16;12:12211. doi: 10.1038/s41598-022-16453-3 (PMC9288512; doi:10.1038/s41598-022-16453-3)
Supplement: Supplementary file 16 — Supplementary Tables. [file 41598_2022_16453_MOESM16_ESM.pdf]

Table S1 The Ab-binding peptides acquired from the phage screening and sequencing

NO.	Group A Peptides	Group B Peptides
1	EGYHHGWIHMPS	LVPSATLLSNTF
2	EGSWVHVYPWVQ	EGYHHGWIHMPS
3	NGKLHIHYGMGY	SPSYASASPSMI
4	YQSVAYFVPHLA	AIMGRFVAIPPH
5	LSGSPGITQKRH	LSHSPAVRHTYQ
6	VHLQTIRMPSDE	LSMKLSLPHRHG
7	MGSSDRLVFDAG	FSKGAGWNELMQ
8	LVRVAGETHPFS	EGSWVHVYPWVQ
9	SYHHASPSRYVH	TPLLGTENPKRY
10	MSNPDHRSGYPT	MYSPAQLNGMSL
11	LTLWGDPRYGVA	YHFPHARLLTVL
12	MVTGMGLQRYTS	QASSSLRPNFSH
13	SGPLIHEYRAPS	MQVGTVFPDKVA
14	VTTSGNNQVQTS	EAMGMYPRLDVF
15	KLLDSSLDLLSL	QRDLLYSPMIYF
16	SSWPLPALVNFR	SCDANCGLHDDT
17	VLSTTSRIGWWM	SSVFLTYQPSYQ
18	GTQIVSELIDTL	ATYAVFPTFALG
19	GSKIPSDPKGSP	HHGSALQFMQMW
20	GLWASNVEFVNT	AVEDHSMSLTME
21	VSRHPINLPLRI	TSNAHGIYLQSE
22	AWRDLLSLMQFD	VLSTTSRIGWWM
23	GSSLGYTLPFSS	AMLPKWSVTFTS
24	ITPHASPKLGVH	LSVTSFSTPFNP
25	SAMSMRGNNYHH	SYHHASPSRYVH
26	LVSAGGTWNRVT	YGRLPSHTLWPF
27	VALTNPPRISLN	LSQSFTPHIHRP
28	MKHQVDHFKGKQ	SPYVHLPTSNDH
29	WDLLSNTGSGLL	VSTRILLPEART
30	SAQVAKLLMIGT	LTSGVTNVHRSF
31	SEMPRWNPAASV	DQTGRFVSESWI
32	VPIWVHVVPDRY	GVLSSATSLTLI
33	AHPGFISPTWMS	SASSANIDLIRL
34	FTSSGTPNPSNL	TCPVCVIRNMPG
35	LTVMGGEKEPKP	HGQLVLVGQGPS
36	LVVHGKDYYVVR	ILPARHELIHRI
37	MHLHSSYDSQSE	LVYVDSAIVNSK
38	RISHQTIPYGGI	SSGSHALFRASH
39	SLSAWNRADKGW	SYHHGLSGTWPS
40	TTAMVGWWMQEV	VSSIKYNRVQNM
41	TYHHGFINSYAR	WIELPKPITMRG
42	VAAIGCCWKVSR	ASTTNLVSRYVL
43	GYMSERWIRWFG	LSIGPTYGQIDA
44	GYPIMYLHTLGR	LSSAPTYTTYWK
45	LSFPQNMRDIPP	MTGPVHANQIRN
46	TLGLSAFNSGLQ	FKMHQPPRQSVS
47	VKIPLLFDVSDR	LSPYIRNNGGIL
48	WTPLRWPALHLN	RAATAPDPATIL
49	YASFGQLADMPN	TGQPPSVRPWTG

50	YKPLPIMQLNWL	VLPTSERPSPSV
51	YRTQNWMAGPHE	AYHHSFTPVPSS
52	FSSGRLTTTIVL	HASYRSPPFPLF
53	ATFASSHTPLTL	LSIPVQPRFGAT
54	DAFSSWAPNYQG	LTLGVAVPTRIL
55	FLPIINFAPHPF	LTVTERITPLSH
56	FWETQPVLAHTR	RAFMEITVYNSA
57	GSSSKTFSQIFR	RVDSVTVLASSI
58	GVFATLLVADGK	TGTMDTATNNTA
59	HVLVVNMKSMTS	YTSSLAVLSSTP
60	KVSGTDVYWRTV	QIIVWSNEVSEM
61	LGGPSRYAMWIN	SVRLPINVVNAP
62	NYHHSWPTLINM	VFSATLNFRHWL
63	QSQSGAQERRME	VRPVSLLISTQI
64	QSVAGSQSLITA	YGLMVPTPFLAR
65	QYPVDLNLQPST	GASRSLPSAVPQ
66	TQPREGASHADI	GLHPWTALVMTM
67	LASHMVEHPRRH	GLRELYVLPLSF
68	MNTARSVELHMS	GLRSDSLSPTLF
69	AFTKLHYIVGSR	GPDKMGWWMNMI
70	CSLRNLTSMLMT	HDGLTWSLETGP
71	CTLSDNMFSCLG	LLLPLNTGRTLL
72	DRGLYTLFTFTL	LSNPGYAMAWKM
73	DYRMMSAMTVLM	LTPTYNYHHGWT
74	EGHVTRLVLSVH	MLVSGDLNALRQ
75	ELAWYADGLYKP	MSGSNSMERLDL
76	FASSTSSIASPS	SAMWRMTSLGQA
77	FHSSLKTYNFSS	SVGVLPLHTLDY
78	FVTPHLLSTNYF	TGPLLLSSTTPA
79	GHITKSCQAAHA	WPRPIGSLPAHS
80	GNNSRENVYLSS	YGAVASMGTPYL
81	GPSIRSITLLSA	VAKAPYVRLSPQ
82	GVFADRWAISSV	GFMSAGVPGVLP
83	HGNRSFDEILTV	AATYSGDGKLGA
84	IVGRLGVNVWLT	AFDGWRVHHRWT
85	KTPAWPLPPFNV	AGAMRHLDGRLY
86	LDRWRVVPQGSH	ANHVEIFTTILR
87	LGHKYYEMKATP	APVRHSSTTYLH
88	LPIMDRVPRLMM	ASQFLGSWESSS
89	LVKMNAPVVLSR	AYSIFNAEMRKN
90	MDVRGTRAVTSS	DGPNEVKLYWSQ
91	MSIEAARNPLAS	DVREAWPSGLVA
92	MSNATVHARLVS	ENMPLGRLINHL
93	MTSQVDSMRLKQ	FPPICCDVLPSK
94	NNSHFIWYPGTT	GGHNDSTFSRDS
95	NVLHAQSDGSIN	GILMYHRGPLFQ
96	QMSAAEWYRTGY	HSVGNMGLGLLS
97	QSSSACGPCAAR	ILSHRPFHPTTA
98	RVLSSPDTPISL	KLPLDGLIHGSV
99	SEFAKAPPAAHS	KPSSYSMSIPTA
100	SPNRSFIESTST	LAPKNSISPGIP

101	SSSSVILFVQHR	LSTFSVVEHWPS
102	SVRNAYGLGNNT	LSTTFPKNLPTS
103	SYDRSENYRNNS	MISGTSARDRNH
104	THSMGPTFVAQA	MYHSKVPMHPNP
105	TIPDEPPNRGLV	NVPRHNYHHSWT
106	TLINQESQYAVF	NWTHPEFDVHRA
107	TPKTIDEATFTL	QLYKSTTSKVLI
108	TSFPISPLAYST	RLAGGNLYDRLP
109	TSNIARMNWYLS	RTEFSSNNGTID
110	TTWRVAPLWSVP	SDHYQGWWMNHL
111	VYPSFALTTLVW	SFHHTWNGLLPD
112	YAAYTRDERLLS	SKGLMDTESYDR
113	YGTARIPAWFGS	SMPVLRYPHIHY
114	YTLKDVRSIQYL	SPSGFWRTLTVD
115	YTVANNLSAVWP	TEFKFKVLGQNA
116		TFRYHMSTALEF
117		TSSYSFVHTSMV
118		VAQDTQRMSGLN
119		VDTQMFMTMDYH
120		VMSPMSISWPLM
121		VTSRIINSWTSW
122		WDAPKPQAALHL
123		YWNGRPPTLTLL
124		FGGAYIHTGINS
125		GGHVHLETTGYI
126		LTRNTPWPEPPR
127		AAKSAYGSPDLY
128		AANLDHAGTYFR
129		AATHGLQRFLHA
130		AFCGRSAPQMTP
131		AFFRLSGPDQLF
132		AFRSSTVPIQLQ
133		AHMGNLDGRWGN
134		ALRAPAHTIWAV
135		ATDMFEVNAGEI
136		AYKCGPLCPSHR
137		AYRIQLDTWVSR
138		DESYFLRSYRFY
139		DKLPSLRKDVGF
140		DPSTTSGGRSVS
141		DVKTLSPPTTSD
142		EFKTIARYFPTR
143		EGSNKAGIRQTW
144		FEVDDLFRLYAI
145		FRVSYGDDHWFR
146		FSSSDLLTVQPL
147		GGDSYIRVTTNR
148		GKVGPATGPNAT
149		GLPKLHYDWSWS
150		GPFSAPSLVPWR
151		GSMSWHMVPRTV

152	GSTVPLDQSSSD
153	GWRHAFSYPVIG
154	HACNTCSRHVAY
155	HEVSWGPHIIHY
156	HIMQSLSAYEGP
157	HNVVTALTQQIW
158	HRAPIVPPLTSP
159	IPAMSRFHATFA
160	IPNSPRPPSMAR
161	ISLSNQTHTYSL
162	KTFTPNHCCSVG
163	LAAPLSTSMFAL
164	LAPHINNLSITM
165	LFYDPVVSGEAG
166	LPQLIQHRAGPD
167	LPVLARDSVSDV
168	LTRHHAPTRLDP
169	MERRLGATYIGM
170	MGLDNTYMRNLD
171	MGPVSPNFSKHK
172	MMCHQMFCGLMA
173	NGDVGMASRVQT
174	NYQIHREVGRIT
175	QFLGRPYLPSHY
176	QHSPVGSPAWAG
177	QNTLTVMMHAHY
178	RLVQDLEGSLSG
179	SGANAFVLHSKA
180	SGSWLSARVAVY
181	SHVSYASGPRFA
182	SHWQHQTLHYTY
183	SLTDWPTSPKVG
184	SSRATTQPIMPS
185	STEGFGWPGHLI
186	STRYPTMGIPTI
187	SVSHALYSLRDV
188	SWFEQFATVLRV
189	SYRYPPFFPLSR
190	SYWTYPGRHIAN
191	TCCWKSHTSADG
192	TGPITATADLSL
193	TGTIHRKLLTPV
194	TPTCTGCPVILL
195	TSPHTQYTQGLL
196	TTHCSPCDMKRF
197	TWGFLPVIYSAG
198	VDSDATKYPAHV
199	VEPRVLNIYGIY
200	VPNMTTHLMPGL
201	VTLSADMFSAQS
202	YHPMGPASVTVW

203	YHTDALVNVPSL
204	YSDHSEWRWTKS
205	YSPHAGPYGRPT
206	NMTYYHTKISHS

Table S2 Identified polypeptides with "GW" similar characteristics

Group	NO.	Peptide	Binding direction
A	1	EGYHHGWIHMPS	F
	2	VLSTTSRIGWWM	F
	3	SLSAWNRADKGW	F
	4	TTAMVGWWMQEV	F
	5	MVTGMGLQRYTS	R
B	1	EGYHHGWIHMPS	F
	2	FSKGAGWNELMQ	F
	3	VLSTTSRIGWWM	F
	4	LSTFSVVEHWPS	F
	5	LTPTYNYHHGWT	F
	6	SDHYQGWWMNHL	F
	7	STEGFGWPGHLI	F
	8	STRYPTMGIPTI	F
	9	HEVSWGPHIIHY	R
	10	TWGFLPVIYSAG	R
	11	TCCWKSHTSADG	R
